# Safety and Metabolism of Long-term Administration of NIAGEN (Nicotinamide Riboside Chloride) in a Randomized, Double-Blind, Placebo-controlled Clinical Trial of Healthy Overweight Adults

**DOI:** 10.1038/s41598-019-46120-z

**Published:** 2019-07-05

**Authors:** Dietrich Conze, Charles Brenner, Claire L. Kruger

**Affiliations:** 1 Chromadex Spherix Consulting, 11821 Parklawn Drive, Suite 310, Rockville, MD 20852 United States; 2 Department of Biochemistry, University of Iowa, 4-403 BSB, Iowa City, IA 52242 United States

**Keywords:** Diagnostic markers, Drug safety

## Abstract

Nicotinamide riboside (NR) is a newly discovered nicotinamide adenine dinucleotide (NAD^+^) precursor vitamin. A crystal form of NR chloride termed NIAGEN is generally recognized as safe (GRAS) for use in foods and the subject of two New Dietary Ingredient Notifications for use in dietary supplements. To evaluate the kinetics and dose-dependency of NR oral availability and safety in overweight, but otherwise healthy men and women, an 8-week randomized, double-blind, placebo-controlled clinical trial was conducted. Consumption of 100, 300 and 1000 mg NR dose-dependently and significantly increased whole blood NAD^+^ (i.e., 22%, 51% and 142%) and other NAD^+^ metabolites within 2 weeks. The increases were maintained throughout the remainder of the study. There were no reports of flushing and no significant differences in adverse events between the NR and placebo-treated groups or between groups at different NR doses. NR also did not elevate low density lipoprotein cholesterol or dysregulate 1-carbon metabolism. Together these data support the development of a tolerable upper intake limit for NR based on human data.

## Introduction

The NAD^+^ co-enzymes NAD^+^, NADH, NADP^+^ and NADPH are the central regulators of metabolism. They are required for fuel oxidation, ATP generation, gluconeogenesis, ketogenesis, production of pentose phosphates, heme, lipids, steroid hormones and detoxification of free radical species^1,2^. NAD^+^ is also a consumed substrate of enzymes that polymerize and/or transfer ADPribose, form cyclic ADPribose (cyclic ADPribose synthetases) and deacylate protein lysine substrates (sirtuins) with production of acyl-ADPribosyl products. Poly(ADPribose) polymerases (PARPs) signal DNA damage in order to assemble repair machinery, while cyclic ADPribose synthetases produce second messengers that mobilize calcium ions from intracellular stores, and sirtuins influence gene expression and protein activities by virtue of reversing protein post-translational modifications^3^. In light of the important roles of NAD^+^ co-enzymes in metabolism and mediating some of the longevity benefits of calorie restriction via sirtuins, there is a renewed interest in the synthesis and maintenance of the NAD^+^ metabolome^4^.

All tissues produce NAD^+^ from nicotinamide (NAM) or the recently identified NAD^+^ precursor, nicotinamide riboside (NR)^5^ Some tissues can produce NAD^+^ from tryptophan *de novo* and nicotinic acid (NA)^2^, although the generation of NAD^+^ from tryptophan is much less efficient than from the vitamin precursors of NA, NAM, or NR, which are collectively termed vitamin B3. NAD^+^ can also be supported by dietary precursors^6^. For example, pellagra, a disease of deficiency of NAD^+^ precursors, can be prevented or treated with approximately 15 mg/day of NA or NAM or with 60-times as much tryptophan^7^. Importantly, despite homeostatic systems and dietary intake of NAD^+^ precursors, it is now known that the levels of NAD^+^ co-enzymes are continuously challenged by metabolic stress. In the overfed and type 2 diabetic mouse livers, levels of NADPH are strikingly depressed^8^, whereas in noise-induced hearing loss^9^, heart failure^10^, peripheral nerve damage^11^, central brain injury^12^ and the liver of a lactating mouse^13^, NAD^+^ levels are compromised. Moreover, NAD^+^ levels have been reported to decline in response to DNA damage^14^, alcohol metabolism^15^, and aging^16,17^, and the expression of nicotinamide phosphoribosyltransferase (NAMPT), the enzyme required for NAM salvage, declines with aging^18^ and chronic inflammation^19^. Thus, considering the relationships between NAD^+^, metabolic stress and aging, nutritional scientists are now investigating whether the ingestion of higher levels of a B3 vitamin should be part of an evidence-based approach to optimize health^2^.

Although NA, NAM, and NR all produce NAD^+^ and NADP^+^^2,7,20^, it is important to note that each precursor has unique effects physiologically. NA can lower blood lipids and is used to treat dyslipidemia^21^. At doses of greater than 50 mg/day, NA can also induce flushing^6,21^. In contrast, NAM does not lower blood lipids or cause flushing, has been reported be a sirtuin inhibitor at high doses^20,22^, and appears to have a greater effect at elevating blood levels of homocysteine (HCY) in humans than NA via its metabolism to 1-methylnicotinamide (MeNAM)^23^. In yeast, NR activates SIR2 and extends replicative lifespan^24^. In mouse models, NR prevents high-fat diet-induced weight gain^25^, fatty liver and diabetic peripheral neuropathy^8^, noise-induced hearing loss^9^, heart failure^10^, and central brain injury^12^. In addition, oral NR greatly improves survival and hematopoietic stem cell regeneration after irradiation of mice—an activity that was not seen in NA or NAM supplemented mice^26^. In rats, oral NR promotes resistance to and reversal of chemotherapeutic neuropathy^27^. In mice, oral NR increases the hepatic levels of the NAD^+^ metabolome with pharmacokinetics that are superior to that of NA and NAM^20^. In addition, postpartum female mice and rats who were administered NR exhibited increased lactation and produced offspring that are stronger, less anxious, have better memory, and have enhanced adult hippocampal neurogenesis and body composition as adults^13^. Because NR does not cause flushing or inhibit sirtuins^25^ and the genes (NRK1 and NRK2) required for the metabolism of NR to NAD^+^ are upregulated in conditions of metabolic stress^10,28^, NR has a particularly strong potential as a distinct vitamin B3 to support human wellness during metabolic stress and aging.

In a variety of animal models, nicotinamide mononucleotide (NMN), the 5′-phosphorylated form of NR, has also shown promise in conditions of metabolic stress and aging^29^. Moreover, the gut-expressed multispanning membrane protein Slc12a8, previously annotated as a Na^+^/K^+^ Cl^−^ transporter, has been proposed to be a specific transporter of nicotinamide mononucleotide (NMN)^30^. However, the assignment of Slc12a8 as a transporter of NMN occurred without a reliable LC-tandem MS assay for the expected concentration of NMN^31^ and are inconsistent with genetic, cell biological, and pharmacological evidence from multiple studies demonstrating that NMN is extracellularly converted to NAM and NR prior to intracellular conversion to NMN and the rest of the NAD metabolome^12,32–36^. While it remains possible that data will emerge showing convincing NMN transport in one or more tissues, the consensus view is that NMN is a usefully circulating metabolite that makes NR available at plasma membranes, which express the 5′-nucleotidase activity of CD73^1,34^. To our knowledge, tests of the safety and human oral availability of NMN are not yet available.

A crystalline form of NR chloride termed NIAGEN has been evaluated in a battery of preclinical studies including a bacterial reverse mutagenesis assay, an *in vitro* chromosome aberration assay, an *in vivo* micronucleus assay, and acute, 14-day and 90-day rat toxicology^37^. In the 90-day toxicology study, NR had a similar toxicity profile to NAM at equimolar doses, the lowest observed adverse effect level (LOAEL) for NR was 1000 mg/kg/day, and the no observed adverse effect level (NOAEL) was 300 mg/kg/day. NIAGEN is Generally Recognized as Safe (GRAS) in the United States for use in food products^38^ and the subject of two new dietary ingredient notifications^39,40^, which were filed with the United States Food and Drug Administration without objection.

To date, NR has also been tested in six clinical trials. The first clinical trial of NR established the safe oral availability of single doses and the timecourse by which NR elevates the human blood NAD metabolome^20^. The second trial provided additional safety data for healthy people taking NR for 8 days^41^. The third and fourth trials addressed NR safety in healthy people either taking 500 mg NR twice daily for 6 weeks or combination of up to 500 mg NR and 100 mg pterostilbene per day for 8 weeks^42,43^. Whereas Dellinger *et al*.^42^ found that the combination of NR and pterostilbene signficantly elevated low density protein cholesterol (LDL-C) in a dose and time-depended fashion^42^, no signficant increases in LDL-C were seen following the adminstration of NR alone^43^. A fifth clinical trial documented the safety and tolerance of ingesting 2 grams NR per day for 12 weeks in obese men and post *hoc* analyses suggested that there was an improvement in fatty liver in the NR-treated group^44^. In a sixth clinical trial, single 500 mg doses of NR depressed markers of oxidative damage while increasing NADPH and exercise performance in older individuals^45^.

To address the dose-dependent oral availability and safety of NR in overweight adults and the safety of daily NR without pterostilbene including effects on LDL-C and blood levels of HCY, we conducted a randomized, 8-week placebo-controlled trial with 3 doses of NR in overweight but otherwise healthy adults. Here we show that once a day doses of NR up to 1 gram per day are safe and orally available. Blood NAD^+^ was increased in study subjects in a dose-dependent manner with NAD^+^ levels achieving 14% to 114% increased levels within 2 weeks that were sustained. We also establish that daily high dose ingestion of NR does not elevate LDL-C or plasma HCY.

## Methods

### Study design

**Figure 1:**
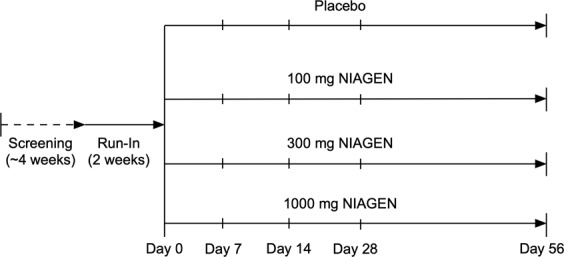
Study design. Subjects were screened over a 4-week period. Eligible subjects were enrolled and instructed to avoid foods containing high amounts of tryptophan and forms of niacin for the duration of the study. Following a 2-week run-in period, the subjects visited the clinic on Day 0 for baseline safety assessments, blood and urine collection, and randomization to one of four supplementation groups (placebo, 100 mg, 300 mg, 1000 mg NIAGEN per day). The subjects then consumed either placebo or the NIAGEN treatments for 56 days and visited the clinic on Day 7, 14, 28, and 56 for safety assessments, and blood and urine collection. Dietary counseling and food records were dispensed and collected throughout the run-in and supplementation periods to ensure that the subjects adhered to the dietary restrictions.

One hundred and forty healthy male and female participants were enrolled in a randomized, double-blind, placebo-controlled parallel study to investigate the safety and effect of NR (100 mg/day, 300 mg/day, and 1000 mg/day) on NAD^+^ metabolite concentrations in urine and blood over 8 weeks. The study consisted of a 2-week run-in and 8-week supplementation period (Fig. 1). To minimize the effect of dietary influences on NAD^+^ metabolite levels, subjects were instructed to avoid foods that contain high amounts of tryptophan and forms of vitamin B3 during the run-in and NR supplementation periods. After screening, all subjects attended the clinic prior to the run-in period to review their medical history and health status and receive counseling for the dietary restrictions. At the end of the run-in period (Day 0), the subjects visited the clinic for baseline safety assessments, blood and urine collection, randomization to one of four supplementation groups (placebo, 100 mg, 300 mg, 1000 mg NIAGEN per day groups; n = 35/group), and additional dietary restriction counseling. The subjects were then released to consume their study product for the subsequent 56 days, attending the clinic on Day 7, 14, 28, and 56 for safety assessments, and blood and urine collection. The study was conducted at KGK Science Inc. Suite 1440, One London Place, 255 Queens Ave, London, Ontario, following Good Clinical Practice (GCP) guidelines and in accordance with the ethical principles that have their origins in the Declaration of Helsinki and its subsequent amendments. The study was reviewed by the Natural Health Product Directorate (NHPD), Health Canada and a research ethics board. Notice of authorization was granted on December 9^th^, 2015 by the NHPD, Ottawa, Ontario and unconditional approval was granted on February 5^th^, 2016 by the Institutional Review Board (IRB Services, Aurora, Ontario). The study was registered on clinicaltrials.org on March 18, 2016 as NCT0271593 and posted to the WHO International Clinical Trial Registry Platform on January 3, 2016. External monitoring of source documents was conducted by ClynProject Consulting, LLC.

The primary objective was to evaluate the difference in urinary MeNAM levels between placebo and NIAGEN (100 mg, 300 mg, and 1000 mg) after 8 weeks of supplementation. The secondary objectives were to evaluate the rate of increase in urinary MeNAM levels between placebo and NIAGEN (100 mg, 300 mg, and 1000 mg) after 8 weeks of supplementation, the difference and rate of increase in other NR metabolites levels in blood between placebo and NIAGEN (100 mg, 300 mg, and 1000 mg) after 8 weeks of supplementation, the difference and rate of increase in other NR metabolites levels in urine between placebo and NIAGEN (100 mg, 300 mg, and 1000 mg) after 8 weeks of supplementation, and the difference in other NR metabolites levels in muscle between placebo and NIAGEN (100 mg, 300 mg, and 1000 mg) after 8 weeks of supplementation. Exploratory outcomes included exploring the changes in Resting Energy Expenditure (REE) relative to placebo after 8 weeks of supplementation, the changes in blood levels of branched-chain amino acids relative to placebo after 8 weeks of supplementation, the changes in blood levels of high sensitivity C-reactive protein (hsCRP) relative to placebo after 8 weeks of supplementation. The safety objectives included the difference in vital signs, hematology and clinical chemistry parameters including high density lipoprotein cholesterol (HDL-C), LDL-C, triglycerides, and total cholesterol between the placebo- and NIAGEN-treated groups, and the difference in the incidence of adverse events between the placebo- and NIAGEN-treated groups. The effect of NIAGEN on plasma HCY levels was determined as a post *hoc* analysis. There were no changes to the trial outcomes or method during the trial and interim analyses were not conducted.

### Subjects

Healthy men and non-pregnant, non-breastfeeding women (40–60 years of age) were eligible for the study if their body mass index was between 25–30, they were willing to avoid vitamin B3 supplements and limit ingestion of foods containing moderate amounts of tryptophan and vitamin B3, maintain current levels of physical activity throughout the study, and refrain from caffeine consumption on days when study visits included blood collection for metabolite measurement. Women of childbearing potential were eligible only if willing to use medically approved forms of birth control. Individuals with diabetes, active peptic ulcer disease, alcohol use >2 standard servings/day or history of drug or alcohol abuse in the past year, using medical marijuana, anti-hypertensives, or lipid lowering medications were excluded. Individuals with a history of renal disease, liver disease, or history of niacin deficiency were also excluded. Individuals were determined healthy by laboratory results, medical exam and physical exam. Informed consent was obtained from each participant at the screening prior to any study-related activities being performed.

### Randomization

The participants were assigned to the different groups by simple randomization. Participants were identified by their initials and their date of birth and were assigned a participant number at their screening visit. If the potential participant met all the inclusion criteria and did not meet any of the exclusion criteria at baseline, a randomization number was assigned to the participant by a blinded investigator per the order of the randomization list generated by www.randomization.com.

### Study product

The study consisted of a 2-week run-in and 8-week interventional period. Participants received either 100 mg, 300 mg, 1000 mg NR per day or placebo during the 8-week intervention. The NR capsule consisted of 100 mg or 250 mg of NR chloride (99% purity) as the active ingredient and microcrystalline cellulose and vegetarian capsule as non-active ingredients. The placebo capsule consisted of microcrystalline cellulose and a vegetarian capsule. No differences in size, color, taste, texture, or packaging were detectable between the two products. The investigational products and the placebo capsules were sealed in identically-appearing blister packets, which were labelled per ICH-GCP and applicable local regulatory guidelines. Unblinded personnel at KGK Science Inc., who were not involved in any study assessments, labelled the investigational product. A randomization schedule was created and provided to the investigator indicating the order of randomization. Investigators, other site personnel, and participants were blinded to the product.

Participants were instructed to take 4 capsules daily after breakfast beginning the day after their randomization visit (Day 1). The 4 capsules amounted to a single dose of either placebo (a total of 4 placebo capsules) or 100 mg NR (1 capsule containing 100 mg NR and three placebo capsules), 300 mg NR (3 capsules containing 100 mg NR and 1 capsule containing placebo) or 1000 mg NR (4 capsules containing 250 mg NR). Participants were instructed to save all unused and open packages and return them at each visit for a determination of compliance. Compliance to the protocol was also assessed by reviewing the 3-day food record and study diaries completed by each participant for adherence to the study’s dietary restrictions, ingestion of the investigational product, and maintenance of physical activity levels.

### Laboratory measurements

Subjects fasted for 12 hours prior to study visits.

Anthropometric measures and vitals were assessed at screening, day 0, 7, 14, 28 and 56. Blood was collected for the assessment of laboratory parameters (CBC, electrolytes Na, K, Cl, HbA1c, creatinine, BUN, AST, ALT, GGT, and bilirubin) at screening, day 0, 7, 14, 28 and 56, blood lipids and NAD^+^ metabolite analyses on day 0, 7, 14, 28, and 56. Urine was also collected for NAD^+^ metabolites analyses on day 0, 7, 14, 28, and 56. The assessments of laboratory parameters and blood lipids were conducted by LifeLabs (Etobicoke, Ontario, Canada) using standardized procedures. NAD^+^ metabolites in blood and urine were quantitated by LC-MS-MS at Keystone Bioanalytical, Inc. (North Wales, PA) using analytically validated methods in accordance with Good Laboratory Practices. Only metabolite data from participants who completed the study and had metabolite levels above the limit of quantitation were included in the analysis.

For whole blood NAD^+^ analysis, NAD^+^-^13^C_5_ was the internal standard. The lower limit of quantification (LLOQ) was 0.3 µg/ml, the upper limit of the quantification (ULOQ) was 50 µg/ml, and the inter-assay precision (% CV) was 1.10 to 11.83%. Plasma NAM was quantified against a NAM-d4 standard with a LLOQ = 5 ng/ml, ULOQ = 3000 ng/ml, and a % CV of 0.71 to 5.38%. Plasma MeNAM was quantified against an MeNAM-d3 standard with a LLOQ = 4 ng/ml, ULOQ = 2000 ng/ml, and a %CV of 0.34 to 13.31%. Urinary MeNAM and N^1^-methyl-2-pyridone-5-carboximide (Me2PY) were quantified against internal d3 standards with an LLOQ = 1 µg/ml and ULOQ = 256 µg/ml for both analytes. The %CV for urinary MeNAM and Me2PY were 1.25 to 4.60% and 1.10 to 3.22%, respectively.

Plasma HCY levels were quantified by LC-MS-MS at Keystone Bioanalytical. Sodium citrate-treated plasma was pretreated with 50 µL of 0.5 M DTT (1,4-dithiothreitol) and HCY and the internal standard (HCY-d4) were precipitated using 0.5% formic acid and 0.05% TFA in acetonitrile. After vortexing and centrifuging, 20 µL of the supernatant was diluted in 200 µL of nano-pure water in a clean HPLC vial, and 5–10 µL was injected into the liquid chromatography mass spectrometer. The standard curve range was 0.2–40 µg/mL with the LLOQ of 0.2 µg/mL.

### Adverse events (AEs)

Subjects were instructed to record any AEs in a diary and were asked at each visit if they have experienced any difficulties or problems since the last visit.

### Statistical analyses

Statistical analyses were completed using the R Statistical Software Package Version 3.2.1 (R Core Team, 2015) for Microsoft Windows. All statistical analyses were performed at a significance level of 5%. Although the primary outcome variable was the difference in urinary MeNAM levels between placebo and NR (100 mg, 300 mg, 1000 mg) treated subjects after 8 weeks of supplementation, the study was powered for a secondary outcome of elevation of blood NAD^+^. Statistical power was based on the estimated standard deviation of 10.1 µM for blood NAD^+^ levels^46^ and 80% power to detect an effect size of at least an 8.7 µM increase. With attrition estimated at 20% throughout the course of the study, a total of 140 subjects were enrolled. For reference, if the study had been powered to detect a significant increase in MeNAM levels, then a total of 128 subjects would have been required.

Statistical analyses were performed on a modified intent-to-treat population (ITT), which consisted of all subjects who received either product, and on whom any post-randomization efficacy information is available. Variables were tested for normality and log-normality where log-normality distributed variables were analyzed in the logarithmic domain. Appropriate non-parametric tests were used to analyze non-normal variables. All missing values were imputed with last observation carried forward (LCOF) imputation. No imputation was performed for missing values of safety variables.

Numerical endpoints were formally tested for significance between groups by analysis of covariance (ANCOVA). The dependent variable was the value at each visit, the factor was the treatment group, and the value at baseline (Day 0) was the covariate. When the effect of supplementation was significant (p-value ≤ 0.05), the pairwise Tukey-Kramer post *hoc* test was applied. Significant efficacy of the product, relative to placebo, was inferred if the coefficient of the treatment group in the ANCOVA model was significantly different from zero (p ≤ 0.05). Numerical endpoints that are intractably non-normal were assessed by the Mann-Whitney U test. A within group analysis on efficacy endpoints was done using the Student’s paired t-test or, in the case of intractable non-normality, the Wilcoxon sign rank test was performed.

## Results

### Compliance and completion of the clinical study

**Figure 2:**
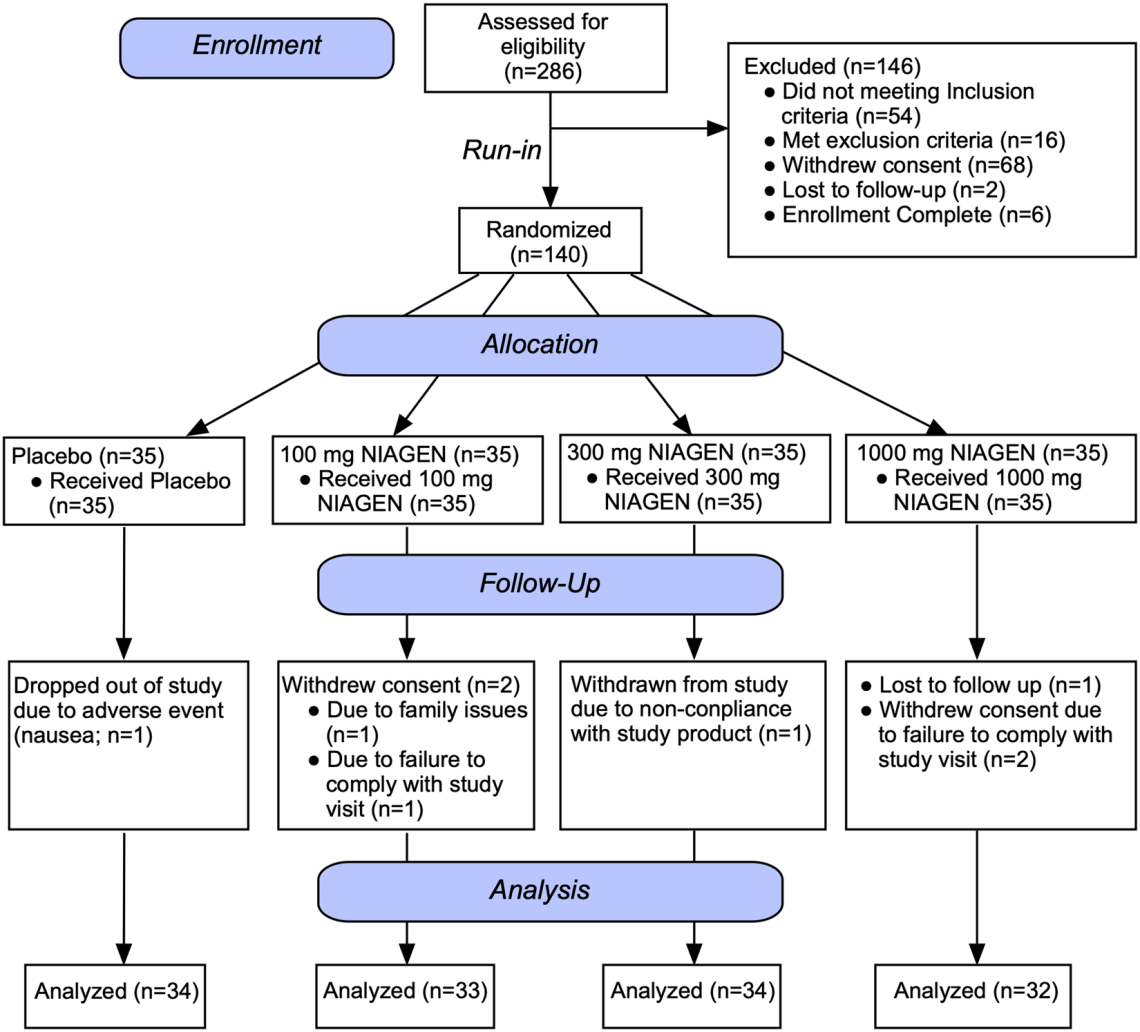
Disposition of the study participants. Two hundred and eighty-six men and women were screened for eligibility. One hundred and forty subjects met the eligibility criteria and were enrolled in the study. After the 2-week run-in (Day 0), the subjects were randomized to one of four treatment groups (Placebo, 100 mg, 300 mg, or 1000 mg NIAGEN per day; n = 35/group). Over the course of the 56-day supplementation period, one subject withdrew from the placebo-treated group due to an adverse event, two subjects withdrew consent in the 100 mg NIAGEN treated group, one subject was withdrawn from the 300 mg NIAGEN-treated group and two subjects withdrew consent and one was lost to follow-up in the 1000 mg NIAGEN-treated group.

Two hundred and eighty-six subjects were screened against the eligibility requirements. One hundred and forty subjects with an age range of 40–60 years and a body mass index of 25.0–30.1 kg/m^2^ were deemed healthy per their screening laboratory values (complete blood panel, hematology, electrolytes, and liver and kidney function tests) and enrolled in the study (Fig. 2). After the 2-week run-in period, subjects were randomized to one of four treatment groups (placebo; 100 mg NR/day; 300 mg NR/day; 1000 mg NR/day). There were no significant differences in any of the screening laboratory values between the different groups. There were also no differences in demographics, anthropometric measurements or vital signs between groups (Table 1). The first potential participant was screened on March 1, 2016 and the last participant’s last visit was on March 17, 2017. The trial was ended after the last randomized subject completed the last visit.

**Table 1:** Demographics of All Participants Enrolled in the Study at Screening.

	Placebo (n = 35)	100 mg NIAGEN (n = 35)	300 mg NIAGEN (n = 35)	1000 mg NIAGEN (n = 35)
**Age (years)**				
Mean ± SD	50.7 ± 5.6	52.3 ± 5.9	50.2 ± 5.8	50.9 ± 5.6
**Gender [n (%)]**				
Male	12 (34%)	12 (34%)	16 (46%)	15 (43%)
Female	23 (66%)	23 (66%)	19 (54%)	20 (57%)
**Alcohol Use [n (%)]**				
None	0 (0%)	1 (3%)	1 (3%)	0 (0%)
Occasionally	12 (34%)	12 (34%)	12 (34%)	19 (54%)
Weekly	17 (49%)	17 (49%)	15 (43%)	10 (29%)
Daily	6 (17%)	5 (14%)	7 (20%)	6 (17%)
**Smoking Status [n (%)]**				
Current Smoker	4 (11%)	3 (9%)	3 (9%)	4 (11%)
Non-Smoker	26 (74%)	19 (54%)	26 (74%)	28 (80%)
Ex-Smoker	5 (14%)	13 (37%)	6 (17%)	3 (9%)
**Race [n (%)]**				
Western European White	28 (80%)	28 (80%)	29 (83%)	29 (83%)
Eastern European White	1 (3%)	1 (3%)	2 (6%)	3 (9%)
Black African American	0 (0%)	1 (3%)	1 (3%)	1 (3%)
East Asian	1 (3%)	1 (3%)	0 (0%)	0 (0%)
South East Asian	0 (0%)	1 (3%)	0 (0%)	0 (0%)
Middle Eastern	1 (3%)	1 (3%)	1 (3%)	0 (0%)
Central American	1 (3%)	0 (0%)	0 (0%)	0 (0%)
South American	3 (9%)	2 (6%)	2 (6%)	2 (6%)
**Ethnicity [n (%)]**				
Hispanic or Latino	5 (14%)	2 (6%)	3 (9%)	2 (6%)
Not Hispanic or Latino	30 (86%)	33 (94%)	32 (91%)	33 (94%)
**Male Serum Creatinine [μmol/L]**				
Mean ± SD (n)	76.4 ± 8.2 (12)	82.5 ± 13.1 (12)	81.0 ± 11.4 (16)	82.7 ± 14.5 (15)
**Female Serum Creatinine [μmol/L]**				
Mean ± SD (n)	64.1 ± 10.6 (23)	62.8 ± 12.5 (23)	65.1 ± 12.5 (19)	61.7 ± 10.4 (20)
**Systolic Blood Pressure (mmHg)**				
Mean ± SD	118.9 ± 10.5	121.6 ± 13.3	123.2 ± 11.5	122.5 ± 14.2
**Diastolic Blood Pressure (mmHg)**				
Mean ± SD	74.5 ± 7.0	75.2 ± 9.1	78.0 ± 8.7	78.4 ± 11.1
**Heart Rate (BPM)**				
Mean ± SD	67.7 ± 9.4	70.2 ± 9.3	66.0 ± 8.2	67.9 ± 8.3
**Weight (kg)**				
Mean ± SD Median (Min − Max)	76.1 ± 7.8	79.5 ± 9.2	79.6 ± 9.8	79.7 ± 8.2
**BMI (kg/m** ^**2**^ **)**				
Mean ± SD	28 ± 2	28 ± 2	28 ± 1	28 ± 2

Kg, kilogram; L, liter; m, meter; Max, maximum; Min, minimum; μmol, micromole**;** n, number; %, percentage; SD, standard deviation.

BPM, beat per minute; kg, kilogram; m, meter; Max, maximum; Min, minimum; mmHg, millimeter of mercury; N/n, number; SD, standard deviation.

Seven participants failed to complete the study (Fig. 2). One subject dropped out of the placebo group due to nausea, one subject was withdrawn from the 300 mg NR-treated group due to non-compliance with the study product, four subjects in the 100 and 1000 mg NR-treated groups withdrew consent (100 mg NR, n = 2; 1000 mg NR n = 2), and one subject in the 1000 mg NR group was lost to follow-up.

Compliance to NR, measured by counting unused capsules returned to the study site, was 98% with a mean compliance of 97.5% in the 100 mg/day NR group, 98.6% in the 300 mg/d group, 97.1% in the 1000 mg/d group, and 99% for participants in the placebo group. Based on dietary records maintained by the subjects, there were no significant between-group differences in total caloric intake or intake of forms of vitamin B3 during the course of the trial.

### NR produces dose-dependent increases in blood and urinary NAD^+^ metabolites

#### Blood NAD^+^

**Figure 3:**
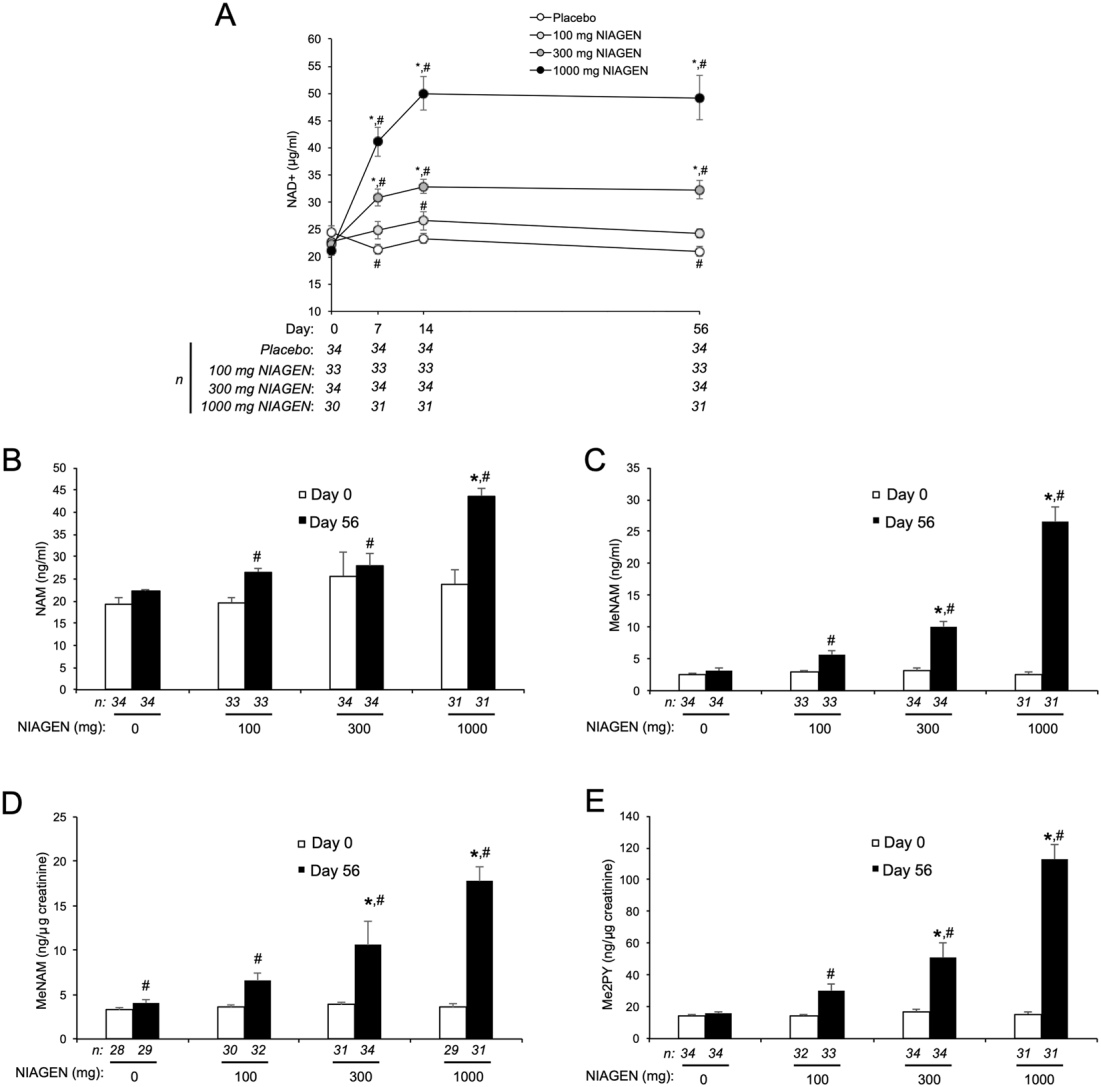
NIAGEN supplementation significantly increases NAD^+^ and other NAD^+^ metabolites. (**A**) Whole blood levels of NAD^+^ in the intent-to-treat (ITT) population over the course of 56 days of placebo, 100, 300, or 1000 mg of NIAGEN per day supplementation. (**B**) Plasma nicotinamide (NAM); (**C**) Plasma 1-methylnicotinamide MeNAM; (**D**) urinary (MeNAM); and (**E**) urinary N-methyl-2-pyridone-3/5-carboximide (Me2PY) levels in the ITT population before and after 56 days of supplementation with placebo, 100, 300, or 1000 mg of NIAGEN per day. Urinary MeNAM and Me2PY levels were normalized to urinary creatinine concentrations. Asterisks denote significant (p < 0.05) between group differences versus placebo. Number signs denote significant (p < 0.05) within group differences relative to Day 0. Error bars represent standard error of the mean. Only data from participants who completed the study and had metabolite levels above the limit of quantitation were included in the analysis. Data for within group differences in panels A, B, C and E were transformed logarithmically to achieve normality.

NAD^+^ levels in peripheral blood mononuclear cells (PBMCs) peak 8 hours after the administration 300 and 1000 mg of NR^20^. However, the time course and dose-dependency by which oral NR increases steady-state NAD^+^ levels in whole blood is not known. Relative to baseline, small but significant decreases in blood NAD^+^ levels occurred in the placebo group over the 56-day supplementation period (p < 0.05). In contrast, daily doses of 300 mg and 1000 mg NIAGEN significantly (p < 0.05) increased NAD^+^ within seven days relative to baseline and placebo (Fig. 3A) and were sustained for the remainder of the study. Blood NAD^+^ levels in the 100 mg-treated group were significantly increased at day 14 relative to baseline and similar to the placebo group at all time points. The day 56 whole blood NAD^+^ level and the rate of change effect sizes also increased dose-dependently to 1.74 and 1.98, respectively (Supplemental Tables 3 and 4). At day 14, the blood NAD^+^ levels of the 100 mg, 300 mg and 1000 mg participants were increased by 22 ± 9%, 51 ± 7% and 142 ± 14% with respect to their baseline blood NAD^+^ levels. At day 56, the blood NAD^+^ levels of the same 100 mg, 300 mg and 1000 mg participants were sustained at increases of 10% ± 4%, 48 ± 8% and 139 ± 19% with respect to their baseline blood NAD^+^ levels.

#### Plasma and urinary metabolites

NAD^+^-consuming enzymes such as the sirtuins, PARP, and cyclic ADPribose synthases hydrolyze the linkage between the NAM and the ADPribosyl moieties of NAD^+^, producing NAM and ADPribosyl products^14,47,48^. NAM then circulates and is methylated in the liver and other tissues to MeNAM^49–51^. Both plasma and urinary blood MeNAM and its oxidation products Me2PY and Me4PY are considered to be biomarkers of increased NAD^+^ metabolism^52^. Fifty-six days of supplementation with NR resulted a significant (p < 0.05) increase in plasma NAM in the 1000 mg group compared to placebo (Fig. 3B) with an effect size of 1.21 (Supplemental Table 5). Relative to baseline, significant (p < 0.05) increases in plasma NAM were also detected in the 100, 300 and 1000 mg-treated groups. Correspondingly, plasma and urinary levels of MeNAM and Me2PY were also significantly (p < 0.05) and dose-dependently increased in the 300 and 1000 mg-treated groups compared to placebo (Fig. 3C–E), resulting in day 56 metabolite level and rate of change effect sizes that ranged from 0.49 to 2.85 and increased with the amount of NIAGEN ingested (Supplemental Tables 1, 2, 7, 8, 9 and 10). Significant (p < 0.05) and dose-dependent increases plasma and urinary levels of MeNAM and Me2PY relative with baseline were also noted in the 100, 300 and 1000 mg groups (Fig. 3C–E).

### Oral NIAGEN is safe and well-tolerated up to 1000 mg/day for 8 weeks

#### No dose-dependent AEs

AEs were coded with Medical Dictionary for Regulatory Activities version 17.0. According to this coding system, flushing (flushing, feeling of warmth transient, hot flush) would be reported under the general disorders and administration site conditions. Ninety-five AEs were reported by 61 participants (Table 2). There were no serious AEs or reports of flushing. Moreover, the type, incidence and severity of the AEs were similar across the different groups.

**Table 2:** Adverse Events and Number of Participants Experiencing at Least One Adverse Event in the ITT Population Separated by Organ Class Category.

Adverse Event	Placebo (n = 35)		100 mg NIAGEN (n = 35)		300 mg NIAGEN (n = 35)		1000 mg NIAGEN (n = 35)	
	Number of AEs	Participants Experiencing AEs	Number of AEs	Participants Experiencing AEs	Number of AEs	Participants Experiencing AEs	Number of AEs	Participants Experiencing AEs
	n	n (%)	n	n (%)	n	n (%)	n	n (%)
Cardiac disorders	0	0 (0.0%)	1	1 (2.9%)	1	1 (2.9%)	1	1 (2.9%)
Gastrointestinal disorders	5	5 (14.3%)	8	7 (20.0%)	8	5 (14.3%)	4	4 (11.4%)
General disorders and administration site conditions	2	2 (5.7%)	6	6 (17.1%)	5	5 (14.3%)	3	2 (5.7%)
Immune system disorders	0	0 (0.0%)	1	1 (2.9%)	0	0 (0.0%)	0	0 (0.0%)
Infections and infestations	6	6 (17.1%)	4	4 (11.4%)	4	4 (11.4%)	5	4 (11.4%)
Injury, poisoning and procedural complications	0	0 (0.0%)	1	1 (2.9%)	0	0 (0.0%)	0	0 (0.0%)
Investigations	1	1 (2.9%)	0	0 (0.0%)	0	0 (0.0%)	0	0 (0.0%)
Metabolism and nutrition disorders	0	0 (0.0%)	1	1 (2.9%)	0	0 (0.0%)	0	0 (0.0%)
Musculoskeletal and connective tissue disorders	1	1 (2.9%)	3	3 (8.6%)	6	5 (14.3%)	5	3 (8.6%)
Nervous system disorders	3	3 (8.6%)	0	0 (0.0%)	3	2 (5.7%)	2	2 (5.7%)
Renal and urinary disorders	1	1 (2.9%)	0	0 (0.0%)	0	0 (0.0%)	0	0 (0.0%)
Respiratory, thoracic and mediastinal disorders	0	0 (0.0%)	0	0 (0.0%)	0	0 (0.0%)	1	1 (2.9%)
Skin and subcutaneous tissue disorders	1	1 (2.9%)	0	0 (0.0%)	0	0 (0.0%)	1	1 (2.9%)
Vascular disorders	0	0 (0.0%)	1	1 (2.9%)	0	0 (0.0%)	0	0 (0.0%)
**Overall Adverse Events**	**20**	**17** (**48.6%)**	**26**	**16** (**45.7%)**	**27**	**14** (**40.0%)**	**22**	**14** (**40.0%)**

AE, adverse event; n, number.

Of the 26 AEs reported in the 100 mg NR group, 24 were reported as being unlikely or not related to the study product. The 2 AEs reported as being possibly related were leg pain and high blood pressure and were mild in intensity. Of the 27 AEs reported in the 300 mg NR group, 25 were reported as being unlikely or not related to the study product. The 2 AEs reported as being possibly related were nausea and muscle pain and were mild in intensity. Of the 22 AE reported in the 1000 mg NR group, 19 were reported as being unlikely or not related to the study product. The 3 AEs reported as being possibly related were sore back, muscle soreness and nausea and were all mild in intensity. Of the 20 AEs reported in the placebo group, 16 were reported as being unlikely to the study product. Of the 4 AEs reported as being possible related, 3 were mild in intensity (rash, raised liver function tests, nausea) and 1 was moderate in intensity (upset stomach). Importantly, all AEs were resolved by the end-of-study.

#### Vital signs

There were no between-group differences in mean systolic blood pressure, mean diastolic blood pressure, mean heart rate or weight. Further, all within-group changes were within normal clinical ranges and were not of clinical significance for this population.

#### Hematology and clinical chemistry

Some differences were observed in the hematology parameters at day 56 (Table 3, Supplemental Figure). Specifically, decreases occurred in the white blood cell count and monocyte count in the placebo-treated group, white blood cell, neutrophil, and lymphocyte counts in the 100 mg-treated group, white blood cell, neutrophil, lymphocyte, monocyte, and basophil counts in the 300 mg-treated group, and the white blood cell, neutrophil, and lymphocyte counts in the 1000 mg-treated group. In contrast, increases in mean corpuscular volume, mean corpuscular hemoglobin, and red cell distribution width occurred only in the 1000 mg-treated group. Statistically significant differences also occurred in the white blood cell count in the 300 mg group compared to the placebo-, 100 mg-, and 1000 mg-treated groups and the red cell distribution width in 1000 mg-treated group compared to placebo-, 100 mg-, and 300 mg-treated groups. Importantly, the differences were not dose-dependent, within the healthy clinical reference ranges for the laboratory and clinic location, and deemed to be not clinically meaningful or an AE.

**Table 3:** Hematology After 56 Days of NIAGEN.

Parameter	Value	Results (Mean ± St. Dev. (n))			
		Placebo^δ^	100 mg NIAGEN^δ^	300 mg NIAGEN^δ^	1000 mg NIAGEN^δ^
Hemoglobin (g/L)^*,§,Δ^	Screening	137.8 ± 9.6 (35)	140.3 ± 12.3 (35)	140.1 ± 14.1 (35)	140.1 ± 13.4 (35)
	Day 56	137.6 ± 9.3 (34)	138.9 ± 12.8 (33)	137.0 ± 11.7 (34)	138.8 ± 13.8 (32)
	Change from screening	−0.6 ± 6.3 (34)	−1.1 ± 6.1 (33)	−2.0 ± 5.7 (34)	−1.2 ± 6.5 (32)
Hematocrit (L/L)^*,§,Δ^	Screening	0.409 ± 0.026 (35)	0.415 ± 0.032 (35)	0.414 ± 0.036 (35)	0.414 ± 0.035 (35)
	Day 56	0.409 ± 0.027 (34)	0.409 ± 0.032 (33)	0.406 ± 0.031 (34)	0.410 ± 0.035 (32)
	Change from screening	−0.0009 ± 0.0175 (34)	−0.0048 ± 0.0177 (33)	−0.0050 ± 0.0162 (34)	−0.0037 ± 0.0170 (32)
White Blood Cell Count (×10^9^/L)^§,Δ^	Screening	6.31 ± 1.21 (35)	6.29 ± 1.63 (35)	6.17 ± 1.45 (35)	6.54 ± 1.90 (35)
	Day 56	5.83 ± 1.25 (34)^a^	5.65 ± 1.68 (33)^a.b^	4.96 ± 1.01 (34)^b^	5.69 ± 1.41 (32)^a.b^
	Change from screening	−0.49 ± 1.13 (34)^a,∞^	−0.59 ± 0.84 (33)^a,∞^	−1.10 ± 1.29 (34)^a,∞^	−0.99 ± 1.23 (32)^a,∞^
Red Blood Cell Count (×10^12^/L)^§,Δ^	Screening	4.59 ± 0.42 (35)	4.64 ± 0.41 (35)	4.74 ± 0.44 (35)	4.71 ± 0.46 (35)
	Day 56	4.60 ± 0.39 (34)	4.60 ± 0.41 (33)	4.68 ± 0.39 (34)	4.63 ± 0.47 (32)
	Change from screening	−0.001 ± 0.197 (34)	−0.045 ± 0.181 (33)	−0.044 ± 0.198 (34)	−0.069 ± 0.202 (32)
Mean Corpuscular Volume (fL)^§,Δ^	Screening	89.4 ± 4.1 (35)	89.6 ± 4.7 (35)	87.7 ± 4.0 (35)	88.2 ± 3.4 (35)
	Day 56	89.3 ± 4.2 (34)	89.5 ± 5.2 (33)	87.1 ± 4.0 (34)	88.7 ± 3.7 (32)
	Change from screening	−0.12 ± 1.98 (34)	0.09 ± 1.42 (33)	−0.32 ± 1.53 (34)	0.50 ± 1.08 (32)^∞^
Mean Corpuscular Hemoglobin (pg)^§,Δ^	Screening	30.10 ± 1.60 (35)	30.28 ± 1.96 (35)	29.57 ± 1.52 (35)	29.80 ± 1.13 (35)
	Day 56	30.00 ± 1.50 (34)	30.23 ± 1.91 (33)	29.31 ± 1.51 (34)	29.97 ± 1.23 (32)
	Change from screening	−0.14 ± 0.67 (34)	0.04 ± 0.58 (33)	−0.14 ± 0.62 (34)	0.19 ± 0.51 (32)^∞^
Mean Corpuscular Hemoglobin Concentration (g/L)^§,Δ^	Screening	336.7 ± 6.7 (35)	338.0 ± 6.9 (35)	337.4 ± 7.5 (35)	338.2 ± 7.3 (35)
	Day 56	336.0 ± 5.7 (34)	338.0 ± 7.0 (33)	336.4 ± 6.7 (34)	337.7 ± 6.6 (32)
	Change from screening	−0.9 ± 6.1 (34)	0.3 ± 5.5 (33)	−0.6 ± 5.6 (34)	−0.1 ± 5.3 (32)
Red Cell Distribution Width (%)^§,Δ^	Screening	13.69 ± 0.71 (35)	13.48 ± 0.54 (35)	13.84 ± 0.81 (35)	13.56 ± 0.55 (35)
	Day 56	13.58 ± 0.74 (34)	13.51 ± 0.52 (33)	13.76 ± 0.69 (34)	13.84 ± 0.73 (32)
	Change from screening	−0.10 ± 0.45 (34)^a^	0.03 ± 0.55 (33)^a.b^	−0.08 ± 0.57 (34)^a.b^	0.25 ± 0.50 (32)^b,∞^
Platelet Count (×10^9^/L)^*, §,Δ^	Screening	265 ± 48 (35)	265 ± 54 (35)	263 ± 43 (35)	276 ± 55 (35)
	Day 56	265 ± 51 (34)	265 ± 63 (33)	252 ± 36 (34)	269 ± 71 (32)
	Change from screening	−2.4 ± 29.4 (34)	−3.2 ± 27.8 (33)	−11.2 ± 27.5 (34)	−9.3 ± 33.0 (32)
Neutrophil Count (×10^9^/L)^*,§,Δ^	Screening	3.63 ± 0.96 (35)	3.52 ± 1.12 (35)	3.68 ± 1.23 (35)	3.83 ± 1.26 (35)
	Day 56	3.34 ± 1.00 (34)	3.11 ± 1.12 (33)	2.78 ± 0.96 (34)	3.17 ± 0.93 (32)
	Change from screening	−0.31 ± 0.96 (34)	−0.41 ± 0.74 (33)^∞^	−0.82 ± 1.22 (34)^∞^	−0.76 ± 0.96 (32)^∞^
Lymphocyte Count (×10^9^/L)^*,§,Δ^	Screening	1.96 ± 0.62 (35)	2.09 ± 0.58 (35)	1.77 ± 0.32 (35)	1.93 ± 0.73 (35)
	Day 56	1.80 ± 0.46 (34)	1.87 ± 0.57 (33)	1.56 ± 0.31 (34)	1.77 ± 0.54 (32)
	Change from screening	−0.14 ± 0.40 (34)	−0.18 ± 0.35 (33)^∞^	−0.19 ± 0.31 (34)^∞^	−0.18 ± 0.38 (32)^∞^
Monocyte Count (×10^9^/L)^†,‡^	Screening	0.523 ± 0.135 (35)	0.483 ± 0.150 (35)	0.511 ± 0.164 (35)	0.523 ± 0.165 (35)
	Day 56	0.485 ± 0.102 (34)	0.545 ± 0.460 (33)	0.441 ± 0.146 (34)	0.491 ± 0.147 (32)
	Change from screening	−0.038 ± 0.107 (34)^∞^	0.067 ± 0.463 (33)	−0.062 ± 0.126 (34)^∞^	−0.038 ± 0.139 (32)
Eosinophil Count (×10^9^/L)^†,‡^	Screening	0.186 ± 0.119 (35)	0.174 ± 0.117 (35)	0.169 ± 0.141 (35)	0.226 ± 0.174 (35)
	Day 56	0.174 ± 0.083 (34)	0.188 ± 0.124 (33)	0.171 ± 0.147 (34)	0.244 ± 0.164 (32)
	Change from screening	−0.018 ± 0.090 (34)	0.024 ± 0.083 (33)	0.000 ± 0.115 (34)	0.006 ± 0.105 (32)
Basophil Count (×10^9^/L)^†,‡^	Screening	0.009 ± 0.028 (35)	0.017 ± 0.038 (35)	0.023 ± 0.043 (35)	0.023 ± 0.043 (35)
	Day 56	0.009 ± 0.029 (34)	0.012 ± 0.033 (33)	0.009 ± 0.029 (34)	0.012 ± 0.034 (32)
	Change from screening	0.000 ± 0.025 (34)	−0.006 ± 0.035 (33)	−0.015 ± 0.036 (34)^∞^	−0.012 ± 0.034 (32)

fL, femtoliter; g, gram; L, liter; Max, maximum; m, meters; μg, microgram; μmol, micromoles; mL, milliliter; mmol, millimoles; Min, minimum; min, minutes; nmol, nanomoles; N, number; % Percent; pg, picogram; SD, standard deviation; U, units.

^§^Between group comparisons were made using ANOVA.

^†^Between group comparisons were made using the Kruskall-Wallis test.

^Δ^Between group comparisons were made using ANCOVA adjusting for screening.

^δ^Within group comparisons were made using the paired Student t-test.

^‡^Within group comparisons were made using the non-parametric signed-rank test.

*Logarithmic transformation was required to achieve normality.

^∞^Denotes statistically significant (p < 0.05) within group differences.

Endpoints with different superscript letters denotes statistically significant (p < 0.05) between group differences via Tukey-Kramer pairwise test.

Recently, dose-dependent, statistically significant increases in total cholesterol and LDL-C were observed in a clinical study in which participants received a combination of 250 mg NR plus 50 mg pterostilbene or a combination of 500 mg NR plus 100 mg pterostilbene for eight weeks^42^. As shown in Table 4, there were no statistically significant differences in the NIAGEN and placebo-groups with respect to any clinical chemistry parameter. Clinical testing of pterostilbene alone indicates that it produces time and dose-dependent increases in human LDL-C^53^ of a magnitude that are a public health concern^54,55^ and are inconsistent with pterostilbene being a sirtuin 1 activator or included as part of a consumer wellness product^56^.

**Table 4:** Clinical Chemistry After 56 Days of NIAGEN.

Parameter	Value	Results (Mean ± St. Dev. (n))			
		Placebo^δ^	100 mg NIAGEN^δ^	300 mg NIAGEN^δ^	1000 mg NIAGEN^δ^
Sodium Concentration (mmol/L)^†,‡^	Screening	142.11 ± 1.97 (35)	141.26 ± 2.48 (35)	141.80 ± 2.06 (35)	141.00 ± 1.81 (35)
	Day 56	140.97 ± 1.59 (34)	140.52 ± 2.43 (33)	140.74 ± 2.30 (34)	140.91 ± 1.63 (32)
	Change from screening	−1.24 ± 2.09 (34)^∞^	−0.64 ± 2.82 (33)	−1.09 ± 2.30 (34)^∞^	−0.16 ± 2.34 (32)
Potassium (mmol/L)^*,§,Δ^	Screening	4.48 ± 0.42 (35)	4.53 ± 0.51 (35)	4.75 ± 0.51 (35)	4.64 ± 0.39 (35)
	Day 56	4.35 ± 0.32 (34)	4.35 ± 0.34 (33)	4.32 ± 0.27 (34)	4.48 ± 0.37 (32)
	Change from screening	−0.14 ± 0.49 (34)	−0.20 ± 0.60 (33)	−0.42 ± 0.59 (34)^∞^	−0.16 ± 0.48 (32)
Chloride (mmol/L)^*,§,Δ^	Screening	105.51 ± 2.25 (35)	105.23 ± 2.85 (35)	106.06 ± 1.70 (35)	104.97 ± 2.29 (35)
	Day 56	103.76 ± 2.55 (34)	104.06 ± 3.69 (33)	104.44 ± 2.34 (34)	104.12 ± 2.43 (32)
	Change from screening	−1.76 ± 2.85 (34)^∞^	−1.00 ± 3.54 (33)	−1.62 ± 2.80 (34)^∞^	−0.91 ± 2.44 (32)^∞^
Creatinine (μmol/L)^*,§,Δ^	Screening	68.3 ± 11.4 (35)	69.5 ± 15.7 (35)	72.4 ± 14.3 (35)	70.7 ± 16.1 (35)
	Day 56	68.6 ± 10.6 (34)	69.2 ± 13.8 (33)	72.8 ± 14.1 (34)	73.3 ± 17.0 (32)
	Change from screening	−0.2 ± 5.7 (34)	1.0 ± 8.3 (33)	0.9 ± 8.4 (34)	3.2 ± 7.9 (32)^∞^
Estimated Glomerular Filtration Rate (mL/min/1.73 m^2^)^§,Δ^	Screening	96.3 ± 12.3 (35)	94.5 ± 14.3 (35)	93.5 ± 12.4 (35)	95.9 ± 13.2 (35)
	Day 56	95.7 ± 12.5 (34)	94.1 ± 12.8 (33)	93.7 ± 13.1 (34)	92.9 ± 12.6 (32)
	Change from screening	−0.0 ± 6.5 (34)	−1.2 ± 9.9 (33)	0.3 ± 7.9 (34)	−2.6 ± 8.6 (32)
Bilirubin (μmol/L)^*,§,Δ^	Screening	8.0 ± 3.5 (35)	8.9 ± 3.4 (35)	8.3 ± 3.2 (35)	8.9 ± 4.1 (35)
	Day 56	10.2 ± 4.0 (34)	10.5 ± 3.5 (33)	9.8 ± 3.9 (34)	9.2 ± 3.2 (32)
	Change from screening	2.3 ± 3.8 (34)^∞^	1.8 ± 2.8 (33)^∞^	1.5 ± 3.5 (34)^∞^	0.3 ± 3.1 (32)
Blood Urea (mmol/L)^*,§,Δ^	Baseline	5.03 ± 1.13 (35)	4.77 ± 1.26 (35)	5.14 ± 1.24 (35)	5.21 ± 1.00 (35)
	Day 56	4.80 ± 1.07 (34)	4.85 ± 1.20 (33)	4.78 ± 0.89 (34)	5.06 ± 1.34 (32)
	Change from baseline	−0.21 ± 0.90 (34)	0.20 ± 1.24 (33)	−0.41 ± 0.91 (34)^∞^	−0.14 ± 1.25 (32)
Aspartate Transaminase (U/L)^*,§,Δ^	Baseline	23.6 ± 6.3 (35)	21.6 ± 4.7 (35)	21.5 ± 3.9 (35)	21.5 ± 4.8 (35)
	Day 56	22.2 ± 6.6 (34)	20.5 ± 4.6 (33)	21.1 ± 3.6 (34)	20.8 ± 5.5 (32)
	Change from baseline	−1.5 ± 4.4 (34)^∞^	−0.7 ± 4.0 (33)	−0.5 ± 3.8 (34)	−1.1 ± 5.2 (32)
Alanine Transaminase (U/L)^*,§,Δ^	Baseline	23.6 ± 10.3 (35)	20.8 ± 6.6 (35)	21.2 ± 7.9 (35)	23.0 ± 10.4 (35)
	Day 56	24.7 ± 11.9 (34)	20.2 ± 7.7 (33)	20.4 ± 6.7 (34)	21.2 ± 8.9 (32)
	Change from baseline	0.9 ± 7.2 (34)	−0.3 ± 5.5 (33)	−0.8 ± 6.6 (34)	−2.6 ± 7.9 (32)^∞^
Gamma-glutamyl transferase (U/L)^†, ‡^	Baseline	24.3 ± 24.6 (35)	18.9 ± 12.6 (35)	21.0 ± 15.3 (35)	21.2 ± 16.5 (35)
	Day 56	26.4 ± 31.0 (34)	17.7 ± 7.5 (33)	19.9 ± 13.0 (34)	28.0 ± 40.3 (32)
	Change from baseline	2.4 ± 7.8 (34)^∞^	0.2 ± 6.0 (33)	−0.9 ± 5.3 (34)	6.3 ± 26.5 (32)
Total Cholesterol (mmol/L)^§,Δ^	Baseline	5.46 ± 0.86 (35)	5.21 ± 0.82 (35)	5.10 ± 0.84 (35)	5.17 ± 0.98 (35)
	Day 56	5.55 ± 0.77 (34)	5.26 ± 0.90 (33)	5.11 ± 0.74 (34)	5.19 ± 0.92 (32)
	Change from baseline	0.07 ± 0.47 (34)	0.06 ± 0.52 (33)	−0.04 ± 0.46 (34)	−0.07 ± 0.42 (32)
Low-density Lipoprotein Cholesterol (mmol/L)^§,Δ^	Baseline	3.33 ± 0.65 (35)	3.16 ± 0.77 (35)	3.12 ± 0.72 (35)	3.18 ± 0.82 (35)
	Day 56	3.37 ± 0.59 (33)^1^	3.08 ± 0.86 (33)	3.18 ± 0.63 (34)	3.15 ± 0.81 (32)
	Change from baseline	0.04 ± 0.40 (33)	−0.06 ± 0.56 (33)	0.02 ± 0.37 (34)	−0.11 ± 0.34 (32)
High-density Lipoprotein Cholesterol (mmol/L)^*,§,Δ^	Baseline	1.46 ± 0.37 (35)	1.45 ± 0.35 (35)	1.39 ± 0.36 (35)	1.38 ± 0.42 (35)
	Day 56	1.48 ± 0.35 (34)	1.56 ± 0.48 (33)	1.40 ± 0.36 (34)	1.42 ± 0.45 (32)
	Change from baseline	0.013 ± 0.167 (34)	0.087 ± 0.254 (33)	0.005 ± 0.160 (34)	0.031 ± 0.199 (32)
Triglycerides (mmol/L)^*,§,Δ^	Baseline	1.45 ± 0.80 (35)	1.33 ± 0.67 (35)	1.29 ± 0.79 (35)	1.33 ± 0.65 (35)
	Day 56	1.56 ± 0.95 (34)	1.37 ± 0.67 (33)	1.17 ± 0.51 (34)	1.38 ± 0.69 (32)
	Change from baseline	0.09 ± 0.57 (34)	0.07 ± 0.31 (33)	−0.13 ± 0.45 (34)	0.03 ± 0.32 (32)

fL, femtoliter; g, gram; L, liter; Max, maximum; m, meters; μg, microgram; μmol, micromoles; mL, milliliter; mmol, millimoles; Min, minimum; min, minutes; nmol, nanomoles; N, number; % Percent; pg, picogram; SD, standard deviation; U, units.

^1^Low-density lipoprotein cholesterol could not be calculated for one participant in this group because their triglyceride level was greater than 4.52 mmol/L.

^§^Between group comparisons were made using ANOVA.

^†^Between group comparisons were made using the Kruskall-Wallis test.

^Δ^Between group comparisons were made using ANCOVA adjusting for screening.

^δ^Within group comparisons were made using the paired Student t-test.

^‡^Within group comparisons were made using the non-parametric signed-rank test.

*Logarithmic transformation was required to achieve normality.

^∞^Denotes statistically significant (p < 0.05) within group differences.

#### NR and plasma homocysteine

**Figure 4:**
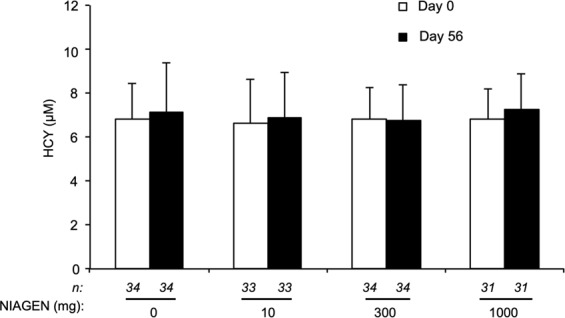
NIAGEN supplementation does not disturb plasma homocysteine. Plasma HCY levels in the intent-to-treat population before and after 56 days of supplementation with placebo, 100, 300, or 1000 mg of NIAGEN per day. Error bars represent standard error of the mean. Only data from participants who completed the study and had metabolite levels above the limit of quantitation were included in the analysis.

Nicotinamide N-methyltransferase catalyzes the transfer of a methyl group from *S*-adenosylmethionine (SAM) to NAM, generating to MeNAM and *S*-adenosylhomocysteine^49–51^. *S*-adenosylhomocysteine is then subsequently cleaved to homocysteine (HCY) and adenosine. It has been reported that single 300 mg oral doses of NA and NAM increase plasma HCY levels^23^, indicating a potential shortage of methyl groups that could be needed for formation of molecules such as dopamine and creatine. Moreover, increased plasma HCY is an independent risk factor for the development of vascular disease^57–59^. To determine whether prolonged ingestion of NR increases plasma HCY levels, a post *hoc* analysis was conducted using sodium citrate-treated plasma samples collected during the study. Compared to baseline or the placebo-treated group, NR ingestion had no effect on plasma HCY levels (Fig. 4).

#### Exploratory endpoints

No significant differences between the any of the NR- and placebo-treated groups were seen in either the REE, blood levels of branched-chain amino acids, or hsCRP after 8 weeks of supplementation.

## Discussion

Because NAD^+^ is the most abundant NAD^+^ metabolite in any cellular sample^60^, it is the breakdown of NAD^+^ and NAD^+^-related coenzymes in food that produces the three salvageable NAD^+^ precursor vitamins: NR, NAM and NA. In addition to the existence of NR in milk^5,61^ and apart from the availability of NR as a supplement, mammals are exposed to NR from the digestive breakdown of dietary NAD^+^ and endogenous NR circulation. Endogenous NR has been shown to be a critical nutrient in maintaining health as mice lacking the major NR kinase gene have depressed hepatic NAD^+^ and depressed liver function^62^. In addition, people undergoing heart failure increase their cardiac expression of the NR kinase 2 gene^10^. This only makes sense if NR is an endogenous form of B3.

NR has been demonstrated to be safe and GRAS, supported by a rigorous battery of animal toxicology studies^37^. Additionally, NR was well-tolerated in all published clinical studies^20,41,43,44^. Because NA use is limited by flushing, it was of particular interest to assess whether there would be reports of flushing or other treatment related AEs that are associated with ingestion of NR. Here we show in a randomized, placebo-controlled, double-blind, parallel-group study involving 140 overweight, otherwise healthy adults that the ingestion of up 1000 mg of NR is not associated with flushing. Limitations of the study were that it was conducted in predominantly white, middle-aged adults who consumed a diet limited in niacin equivalents.

The concept of niacin equivalence among the NAD^+^ precursors is clearly useful when defining reference intakes because adequate amounts of tryptophan, NAM or NA can prevent pellagra^7^. However, niacin equivalency does not apply at the higher doses used to support other health endpoints as evidenced by the independent ULs for NAM and NA derived by the European Commission and UK Expert Group on Vitamins and Minerals. The UL for NA was established at 10 mg/day based on flushing^63^ and the UL for NAM is 900 mg/day based on the NOAELs established in clinical studies administering doses up to 3 g NAM per day^64^. Additionally, on the basis of elevating HCY, a sensitive biomarker of methylation status, NAM and NA differ in terms of their potential to dysregulate 1-carbon metabolism. While both of the classical forms of B3 elevated plasma HCY after single doses of 300 mg, NAM elevated HCY substantially more than NA^23^. On a molar basis, 300 mg of NAM (MW = 122 Da) is equivalent to 716 mg of NR Cl (MW = 291 Da) and our data show that NR does not elevate HCY at daily doses up to 1000 mg for 8 weeks.

NR, NAM and NA are converted to NAD^+^ through three different gene-encoded pathways that are tissue-restricted in the case of NA^2^. Because NA uniquely produces flushing, there is a reason for a lower UL for NA. Additionally, although NAM does not appear to produce AEs, there is some concern around its use as a vitamin due to its ability to dysregulate 1-carbon metabolism^23^ and inhibit sirtuins at high doses^20,22^. The safe oral availability of NR and its lack of adverse effects on HCY and LDL-C at doses up to 1000 mg/day support the establishment of a UL for NR that is equal to or greater than that of NAM.

## Supplementary information

Final Protocol

Supplementary information

## Data Availability

The datasets generated during and/or analyzed during the current study are available by request.
