# Supplementary material for: Safety and Metabolism of Long-term Administration of NIAGEN (Nicotinamide Riboside Chloride) in a Randomized, Double-Blind, Placebo-controlled Clinical Trial of Healthy Overweight Adults
Source: Sci Rep. 2019 Jul 5;9:9772. doi: 10.1038/s41598-019-46120-z (PMC6611812; doi:10.1038/s41598-019-46120-z)
Supplement: Supplementary file 1 — Final Protocol [file 41598_2019_46120_MOESM1_ESM.pdf]

KGK Science

DATE: February 15, 2019

PROTOCOL: 15NRHC

REGARDING: Processing Samples for NAD

NOTE:

A deviation from the protocol was made on the matrix used for NAD analysis. NAD was analyzed from whole blood instead of RBCs. The analytical laboratory, Keystone Bioanalytical Inc. confirmed that the method for the analysis was compatible and valid for whole blood.

Saghar Aghili
Laboratory Manager
KGK Science London
Suite 1440, One London Place
255 Queens Avenue
London, ON N6A 5R8
Phone:519-858-8359
Fax:519-438-8314
Email:saghili@kgkscience.com

CLINICAL PROTOCOL COVER PAGE

**Protocol Title:**	A randomized, double-blind, placebo controlled parallel study investigating the effects of Niagen™ (Nicotinamide Riboside) on Niagen™ metabolites in healthy adults
**Protocol Number:**	15NRHC
**Protocol Date:**	March 03, 2016
**Amendment(s):**	
**Study Phase:**	Phase II
**Study Design:**	Randomized, double-blind, placebo controlled parallel
**Sponsor:**	ChromaDex<br>10005 Muirlands Blvd. Suite G,<br>Irvine CA, 92618<br>USA<br>949-419-0288
**Sponsor Contact:**	Ryan Dellinger, PhD<br>Director, Scientific Affairs<br>ChromaDex<br>949-600-9737
**CRO:**	KGK Synergize Inc.<br>Suite 1440, One London Place<br>255 Queens Ave<br>London Ontario N6A 5R8<br>Canada<br>519-438-9374
**Medical Director:**	Gordon Schacter, MD<br>KGK Synergize Inc.<br>519-438-9374

Protocol 15NRHC: A randomized, double-blind, placebo controlled parallel study investigating the effects of Niagen™ (Nicotinamide Riboside) on Niagen™ metabolites in healthy adults

PROTOCOL SIGNATURE SHEET

Name	Signature	Date
**Sponsor:**<br>Ryan Dellinger, PhD<br>Director, Scientific Affairs<br>ChromaDex<br>10005 Muirlands Blvd., Suite G<br>Irvine, CA 92618<br>U.S.A.	Ryan Dellinger	3-3-16
**Scientific Director:**<br>M. Evans, PhD<br>Scientific Director<br>KGK Synergize Inc.<br>255 Queens Ave.,<br>Suite 1440<br>London, ON N6A 5R8<br>Canada	Malkanthi Evans	March 04, 2016

LIST OF ABBREVIATIONS AND SYMBOLS

AE	adverse event
ALT	alanine transaminase
AST	aspartate aminotransferase
BMI	body mass index
°C	degree Celsius
CBC	complete blood count
Cl	chloride
cm	centimetre
EDTA	diaminoethanetetraacetic acid
e.g.	for example
et al	and others
g	gram
GCP	Good Clinical Practice
GGT	gamma-glutamyltransferase
i.e.	that means
lbs	pounds
ICH	International Conference of Harmonization
IEC	Independent Ethics Committee
IRB	Institutional Review Board
K	potassium
kg	kilogram
L	liter
m	meter
mg	milligram
ml	milliliter
Na	Sodium
NA	nicotinic acid
NAD+	nicotinamide adenine dinucleotide
NR	nicotinamide riboside
RBC	red blood cells
REE	resting energy expenditure
RMR	resting metabolic rate
SAE	serious adverse event
SOP	standard operating procedure
SST	serum separating tube
TPD	Therapeutic Products Directorate
ULN	upper limit of normal
WBC	white blood cell

TABLE OF CONTENTS

LIST OF ABBREVIATIONS AND SYMBOLS 3

1 INTRODUCTION 6

2 STUDY OBJECTIVES 8

3 STUDY DESIGN 9

4 SELECTION OF STUDY POPULATION 10

4.1 INCLUSION CRITERIA 10

4.2 EXCLUSION CRITERIA 10

4.3 CONCOMITANT MEDICATIONS 11

4.4 EARLY WITHDRAWAL 11

5 INVESTIGATIONAL PRODUCT 12

5.1 MANUFACTURING AND STORAGE 12

5.2 LABELING AND CODING 12

5.3 INVESTIGATIONAL PRODUCTS 12

5.4 PLACEBO: 13

5.5 DIRECTIONS 13

5.6 RANDOMIZATION 13

5.7 UNBLINDING AND ALLOCATION CONCEALMENT 13

6 STUDY ASSESSMENTS 13

6.1 VISIT 1 - SCREENING (DAY -28 TO DAY -16) 14

6.2 STABILIZATION INITIATION (DAY -14) 14

6.3 VISIT 3 – BASELINE (DAY 0) 15

6.4 VISIT 4, 5, AND 6 (DAYS 7, 14, AND 28) 16

6.5 VISIT 7- END OF STUDY (DAY 56) 17

6.6 CLINICAL ASSESSMENTS AND PROCEDURES 17

6.6.1 Height, Weight 17

6.6.2 Blood Pressure 18

6.6.3 Micro-Needle Muscle Biopsy 18

6.6.4 Indirect Calorimeter Analysis 18

6.6.5 Compliance 18

6.7 LABORATORY ANALYSES 19

6.8 TERMINATION OF THE TRIAL 21

6.9 PROTOCOL AMENDMENTS 21

7 SAFETY INSTRUCTIONS AND GUIDANCE 21

7.1 ADVERSE EVENTS AND LABORATORY ABNORMALITIES 21

7.1.1 Adverse Events 21

7.1.2 *Serious Adverse Event* ... 22

7.1.3 *Unexpected Adverse Reaction* ... 22

7.1.4 *Laboratory Test Abnormalities* ... 23

7.2 Treatment and Follow-up of AEs and Laboratory Abnormalities ... 23

7.2.1 *Treatment and Follow-up of AEs* ... 23

7.2.2 *Treatment and Follow-up of Laboratory Abnormalities* ... 23

7.3 Reporting of SAEs and Unexpected Adverse Reactions ... 23

7.4 Dietary monitoring ... 24

8 STATISTICAL EVALUATION ... 24

8.1 Determination of sample size ... 24

8.2 Analysis Plan ... 24

8.3 Analysis Plan ... 25

8.3.1 *Premature Discontinuation Description* ... 25

8.3.2 *Safety* ... 25

8.4 Protocol Deviation Description ... 26

8.5 Protocol Amendments ... 26

9 DATA COLLECTION AND STORAGE ... 26

10 ETHICAL ASPECTS OF THE STUDY ... 26

10.1 IRB Approval ... 26

10.2 Subject Information and Informed Consent ... 26

10.3 Potential Risks and Procedures to Minimize Risk ... 27

11 QUALITY ASSURANCE AND QUALITY CONTROL ... 27

11.1 Auditing ... 27

11.2 Monitoring ... 27

11.3 Data Management ... 27

12 REFERENCES ... 28

13 APPENDICES ... 30

13.1 Appendix 1 Schedule of Assessments ... 30

13.2 Appendix 2 Foods to Avoid/Limit during the Study ... 32

13.3 Appendix 3 Reference Range for Serum Amino Acids ... 34

1 INTRODUCTION

Precursors to nicotinamide adenine dinucleotide (NAD+), most notably as nicotinic acid (NA, vitamin B3 or niacin), have been shown to be advantageous for the maintenance of heart health and longevity (Carlson & Rosenhamer, 1988; Karpe & Frayn, 2004). Unfortunately, NA supplementation has been reported to cause flushing via activation of the GPR109A receptor (Benyo et al., 2005) and this side-effect has reduced its efficacy, as well as provided a rational to find alternative primers for NAD+.

Niagen™ is a commercially-available form of nicotinamide riboside (NR), a NAD+ precursor that does not activate the GPR109A receptor (Chi & Sauve, 2013). Dietary sources of NR are found in yeast extracts, such as beer, as well as in dairy products. Importantly, NAD+ acts as a rate-limiting step for NAD+-consuming enzymes, including sirtuins, poly-ADP ribose polymerases (PARPs), and CD38/157 ectoenzymes, which collectively influence mitochondrial metabolism and cell longevity (reviewed in (Imai & Guarente, 2014)). Interestingly, NAD+ levels have been reported to decrease as we age and this may be associated with defects in mitochondrial signalling, which in turn may accelerate the incidence of age-associated pathologies (Imai & Guarente, 2014). Thus, restoring cellular NAD+ levels with precursors such as NR (i.e. Niagen™) have to potential to reverse these age-associated functional defects, as well as enhance cellular energy production via increased mitochondrial biogenesis. Importantly, urinary methylnicotinamide levels parallel cellular NAD concentrations.

A recent study by Canto *et al.*, (2012) found that synthetically derived NR caused an increase in NAD^+ in mouse muscle and liver (Canto et al., 2012). These authors reported that mice challenged with high fat diets did not gain weight and used oxidation of fatty acids as a fuel source and improved insulin sensitivity. Moreover, NR was shown to increase mitochondrial biogenesis in muscle tissue and therefore enhanced the endurance performance of these animals (Canto et al., 2012). Furthermore NR fed mice on either control diets or high fat diets showed increased capacity to maintain body temperature during cold exposure, suggesting an improvement in brown adipose tissue oxidative performance (Canto et al., 2012).

There are numerous proof-of-concept *in vitro* and *in vivo* studies (Bogan & Brenner, 2008; Canto et al., 2012; Khan et al., 2014) that indicate that NR supplementation has beneficial biological effects. However, to date there are no published reports on the safety and bioavailability of NR treatment in humans. We recently conducted a randomized, double-blind, cross-over study to examine the pharmacokinetic effects of Niagen™ dosing in healthy adults ((14NBHC, 2015), unpublished findings). Niagen™ was found to be readily absorbed and exhibit a dose-dependent relationship in human serum and urine. In addition, Niagen™ was well tolerated and presented no toxicity within the acute time-points (1-24h) of the trail. The aim for this study is to investigate the long-term effects of Niagen™ supplementation on Niagen™ metabolite concentrations in blood, urine, and muscle of healthy subjects. Importantly, because urinary N-mehtylnicotinamide levels are considered the most reliable biomarker for niacin supplementation (Jacob et al., 1998;

Institute of Medicine, 1998), urinary N-methylnicotinamide levels will used as the primary endpoint for assessing Niagen™ metabolism .

2 STUDY OBJECTIVES

The objective of this study is to evaluate the effect of repeated doses of Niagen™ (Nicotinamide Riboside, NR) on Niagen™ metabolite concentrations in blood, urine, and muscle in healthy adults.

Primary outcome:
The difference in urinary methylnicotinamide levels between placebo and Niagen™ (100mg, 300mg, and 1000mg) treated subjects after 8 weeks of supplementation.

Secondary outcomes:
1. The rate of increase in urinary methylnicotinamide levels between placebo- and NR (100mg, 300mg, and 1000mg)-treated subjects after 8 weeks.
2. The difference and rate of increase in other NR metabolites (see section 6.7) levels in blood between placebo- and NR (100mg, 300mg, and 1000mg)-treated subjects after 8 weeks.
3. The difference and rate of increase in other NR metabolites (see section 6.7) levels in urine between placebo- and NR (100mg, 300mg, and 1000mg)-treated subjects after 8 weeks.
4. The difference in other NR metabolites (see section 6.7) levels in muscle between placebo- and NR-(100mg, 300mg, and 1000mg)-treated subjects after 8 weeks

Exploratory outcomes
1. Change in Resting Energy Expenditure (REE) relative to placebo after 8 weeks of supplementation
2. Changes in blood levels of branched amino acids relative to placebo after 8 weeks of supplementation
3. Changes in blood levels of high sensitivity C-reactive protein (hsCRP) relative to placebo after 8 weeks of supplementation

Safety outcomes:
1. The difference in vital signs, hematology and clinical chemistry parameters including HDL, LDL, triglycerides, and total cholesterol between placebo and Niagen™.
2. The difference in the incidence of adverse events between the placebo and Niagen™.

3 STUDY DESIGN

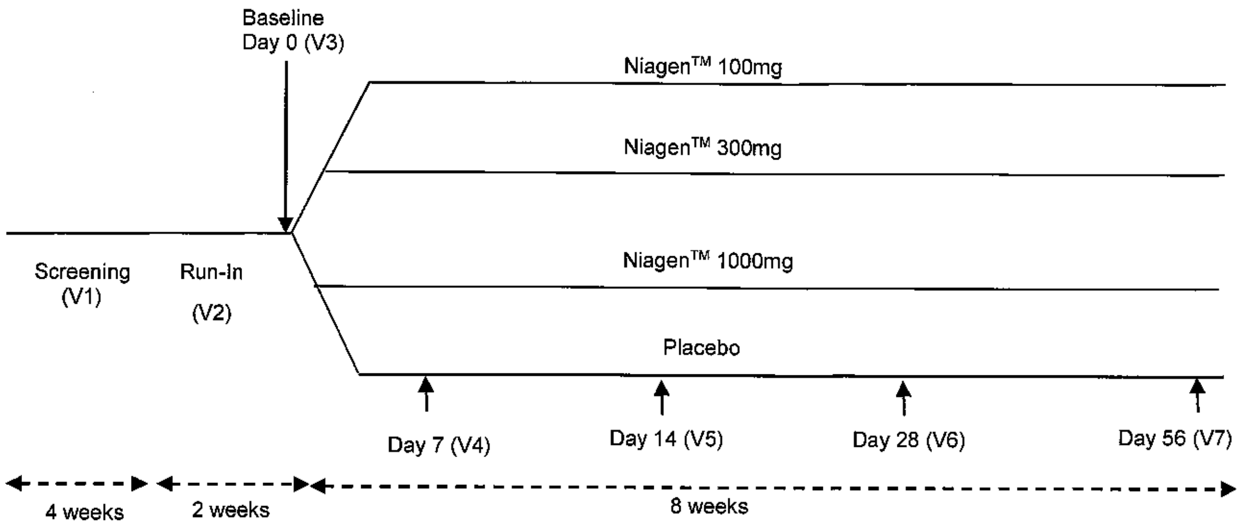

This will be a randomized, double blind, placebo controlled, 4-arm parallel study that will investigate the effect of Niagen™ supplements in healthy subjects on Niagen™ metabolite concentrations in urine, blood, and muscle in healthy males and females.

The planned sample size for this study is 140 healthy males or females, with 35 subjects randomized equally to each of the four study arms in a double-blind manner at a ratio of 1:1:1:1.

**Study Arm**	**Subject Number**
Niagen™ 100mg	N = 35
Niagen™ 300mg	N = 35
Niagen™ 1000mg	N = 35
Placebo	N = 35
**Total**	**N = 140**

In order to evaluate primary, secondary, exploratory, and safety outcomes, study assessments will be conducted at baseline and all study visits. At each visit to the clinic, whole blood (which will be centrifuged and separated into WBC, RBC and plasma fractions) and urine will be collected from each participant for NR metabolite and creatinine analysis. REE will also be calculated at baseline and all subsequent clinic visits using indirect calorimetry. A muscle biopsy will be collected at baseline and end of study (week 8).

The study will be conducted at a single site in London, Ontario.

4 SELECTION OF STUDY POPULATION

This study will include 140 healthy adult volunteers. Each volunteer will have to fulfill the inclusion criteria and not meet any of the exclusion criteria as described in sections 4.1 and 4.2.

4.1 Inclusion Criteria

1. Healthy Male or female 40-60 years of age
2. BMI 25-30 kg/m^2
3. If female, subject is not of child bearing potential, which is defined as females who have had a hysterectomy or oophorectomy, bilateral tubal ligation or are post-menopausal (natural or surgically with > 1 year since last menstruation)

   OR

   Females of childbearing potential must agree to use a medically approved method of birth control and have a negative urine pregnancy test result. Acceptable methods of birth control include:

   - Hormonal contraceptives including oral contraceptives, hormone birth control patch (Ortho Evra), vaginal contraceptive ring (NuvaRing), injectable contraceptives (Depo-Provera, Lunelle), or hormone implant (Norplant System)
   - Double-barrier method
   - Non-hormonal intrauterine devices
   - Vasectomy of partner
4. Healthy as determined by laboratory results, medical history, and physical exam
5. Agrees to comply with study procedures
6. Agrees to maintain current level of physical activity throughout the study and avoid exercising on the day of study visits 3 to 7 (prior to the visit).
7. Agrees to refrain from consuming caffeine (i.e. coffee) on the days of study visits 3 to 7 (prior to the visit)
8. Agrees to avoid taking Vitamin B3 (niacin, nicotinic acid,niacinamide) supplements or multivitamins 14 days prior to randomization and for the duration of the study period
9. Has given voluntary, written, informed consent to participate in the study
10. Agrees to avoid foods that contain high amounts of tryptophan and niacin and limit their ingestion of foods containing moderate amounts of tryptophan and niacin.

4.2 Exclusion Criteria

1. Women who are pregnant, breastfeeding, or planning to become pregnant during the course of the trial.
2. Alcohol use >2 standard alcoholic drinks per day
3. History of alcohol or drug abuse within the past year
4. Medicinal use of marijuana
5. Diabetes (Type I or Type II)
6. Active peptic ulcer disease
7. Subjects taking lipid lowering drugs and blood pressure medications
8. History of renal and/or liver disease
9. History of pellagra or niacin deficiency

10. Significant or untreated medical disorders including recent myocardial ischemia or infarction, unstable angina, uncontrolled hypertension, AIDS, malignancy, and neurological disorders including epilepsy, and recent cerebrovascular disease
11. Subjects with or who had recently experienced a traumatic injury, inflammation, infections or had undergone surgery.
12. Use of natural health products containing NR within 14 days prior to randomization and during the course of the study
13. Unstable medical conditions as determined by the Qualified Investigator
14. Clinically significant abnormal lab results at screening (e.g. AST and/or ALT > 2 x ULN, and/or bilirubin > 2 x ULN) will be assessed by the Medical Investigator
15. History of or current diagnosis of any cancer (except for successfully treated basal cell carcinoma) diagnosed less than 5 years prior to screening. Subjects with cancer in full remission more than 5 years after diagnosis are acceptable
16. Participation in a clinical research trial within 30 days prior to randomization
17. Allergy or sensitivity to study supplement ingredients
18. Allergy or sensitivity to lidocaine
19. Bleeding disorder
20. Serum creatinine ≥ 130μmol/L for males and ≥ 125μmol/L for females
21. Individuals who are cognitively impaired and/or who are unable to give informed consent.
22. Any other condition which in the Investigator's opinion may adversely affect the subject's ability to complete the study or which may pose significant risk to the subjects

4.3 Concomitant Medications

Participants who are currently taking any prescribed medications must agree to maintain their current method and dosing regimen during the course of the study.

Use of blood pressure and cholesterol lowering medications are not permitted.

Use of natural health products/dietary supplements containing niacin (nicotinic acid) or nicotinamide within 14 days of randomization or during the study is not permitted.

4.4 Early Withdrawal

Personal reasons

As stated in the Informed Consent Form, a participant may withdraw from the study for any reason at any time.

Removal by PI:

Participant discontinuation should be considered at the discretion of the Qualified Investigator. The circumstances of any discontinuation have to be documented in detail in the participant file and final report. If possible, the evaluations planned for the end of treatment will be carried out at the time when the participant is withdrawn from the study. A participant leaving the study prematurely will NOT be replaced by another. It is understood by all concerned that an excessive rate of withdrawals can render the study un-interpretable; therefore, unnecessary withdrawal of participants should be avoided.

Criteria for removal of participants from the study will include:

Clinical reasons

A participant may be withdrawn from the study if, in the opinion of the Qualified Investigator, it is not in the participant's best interest to continue. Any participant who experiences a serious adverse event (SAE) may be withdrawn from the trial at the discretion of the Qualified Investigator. A participant will also be withdrawn due to adverse events causing clinically significant illness or the need for prohibited medication(s) during the trial. Any female subject who becomes pregnant during the course of the trial will be withdrawn.

Protocol violation

Any participant found to have entered this study in violation of the protocol will be discontinued from the study at the discretion of the Qualified Investigator. This will include any participant found to have been inappropriately enrolled (did not meet eligibility criteria). Participant non-compliance includes not showing up for study visits, not taking the investigational product as directed, or refusing to undergo study visit procedures. Participants who are found to be taking prohibited medications or supplements without the knowledge of the Qualified Investigator will also be withdrawn. Any major protocol deviations (i.e., those that increase the risk to participants and/or compromise the integrity of the study or its results) will result in participant discontinuation.

5 INVESTIGATIONAL PRODUCT

5.1 Manufacturing and Storage

The investigational product will be provided to KGK by the Sponsor. The investigational product will be carefully stored at the study site in a lockable, limited access area, accessible only to study team personnel in compliance with pertinent regulations. Only authorized persons will have access to the investigational product. The products will be stored at room temperature and will not be exposed to direct sunlight or heat. The investigational products will be kept in a locked investigational product storage room at KGK Synergize Inc. on receipt. An accountability log will be kept for the investigational products.

All unused investigational product will be returned to the study sponsor by KGK (at the sponsor's expense) or destroyed on receipt of written confirmation from the sponsor at study closeout (within one month of last subject visit).

Manufactured by:

W.R. Grace & Co. 1290 Industrial Way, Albany, OR, 97322

5.2 Labeling and Coding

The investigational product will be labeled according to the requirements of ICH-GCP guidelines and applicable local regulatory guidelines. Investigational product will be randomized and coded by an unblinded person at KGK who is not involved in data collection or analysis.

5.3 Investigational Products

Dietary Ingredient	Quantity (Qty)
Niagen™ (nicotinamide riboside chloride)	100mg (1 capsule @ 100 mg)
Niagen™ (nicotinamide riboside chloride)	300mg (3 capsules @ 100 mg)

Niagen™ (nicotinamide riboside chloride)	1000mg (4 capsules @ 250 mg)

Non-medicinal ingredients: microcrystalline cellulose, veggie capsule

5.4 Placebo:

microcrystalline cellulose, veggie capsule

5.5 Directions

Participants will consume 4 capsules at the clinic (Day 0) after acquiring biological samples (blood, urine and muscle) and evaluating REE. Participants will be instructed to take 4 capsules daily after breakfast beginning the day after randomization visit (Day 1). . The 4 capsules amount to a single dose of either placebo (a total of 4 placebo capsules) or 100mg NR (1 capsule containing 100 mg Niagen™ and three placebo capsules), 300mg NR (3 capsules containing 100 mg Niagen™ and 1 capsule containing placebo) or 1000mg (4 capsules containing 250 mg Niagen™). These capsules will be supplied in blister packs (4 per pack), whereby participants will open one blister pack daily to obtain the correct dose. If a dose is missed the participant should take the missed dose as soon as remembered. Participants are not to take more than 4 capsules per day. Clinic staff will also be instruct the participants to save all unused and open packages and return them to KGK at each visit for a determination of compliance.

5.6 Randomization

A randomization schedule will be created and provided to the Investigator indicating the order of randomization. Each participant will be assigned a randomization code according to the order of the randomization list generated using www.randomization.com. Enrolled participants will be block randomized to the different treatment arms at Day 0.

5.7 Unblinding and Allocation Concealment

Unblinding should not occur except in the case of emergency situations. In the event that a serious adverse event occurs, for which the identity of the investigational product administered is necessary to manage the participant's condition, the treatment received by the subject will be unblinded and the investigational product identified. Concealment of the allocation of treatment will be employed through the use of opaque sealed envelopes, each labeled with a randomization number. Each envelope will contain information regarding the treatment associated with each randomization number. These envelopes will be readily available for the investigator to open in the event that it becomes necessary to know which product a participant is taking for the sake of their health care. The sponsor must be notified of any unblinding within 24 hours. Details of participants who are unblinded during the study will be included in the Final Report.

6 STUDY ASSESSMENTS

See Appendix 1 for the schedule of assessments and procedures.

6.1 Visit 1 - Screening (Day -28 to Day -16)

At screening, a Subject Informed Consent Form will be given to the potential subject. The subject will read the information carefully and will be given the opportunity to seek more information if needed. The subject will also be provided with the option of taking the consent form home to review prior to making his or her decision. If agreeable, the subject will sign the consent form and receive a duplicate. Once consent has been obtained, the screening visit will proceed. After the subject has signed the informed consent, the screening number will be assigned sequentially and entered in the Screening and Enrollment Log. Screening numbers will be allocated in the chronological order of the subject's signing the informed consent.

Visit 1 includes:

- Review inclusion and exclusion criteria
- Review of medical history and concomitant therapies and current health status
- Seated resting blood pressure, and heart rate
- Weight and height measured and BMI calculated
- Collect blood samples for CBC, electrolytes (Na, K, Cl), HbA1c, creatinine, AST, ALT, GGT, BUN and bilirubin
- Urine pregnancy test for all female participants

Subjects will be asked to maintain current physical activity levels and record physical activity in a diary (dispensed at visit 2)

Subjects will be educated on the dietary restrictions required for the study by a dietician. These restrictions include:

1. Avoid foods containing high amounts of tryptophan and niacin
2. Limiting exposure to food containing moderate amounts of tryptophan and niacin (refer to appendix 2);
3. Avoiding foods and beverages known to contain NR during specified periods of time prior to and during the study. A list of these foods and beverages are provided below:
    - Food containing niacin (during the study)
    - Vitamins and health supplements containing niacin (nicotinic acid) or nicotinamide for 14 days prior to enrolment and during the study
    - Nutritional yeast (14 days prior to enrolment, during the study)
    - Whey proteins (14 days prior to enrolment, during the study)
    - Energy drinks (14 days prior to enrolment, during the study)

See Appendix 2 for a detailed list of food to avoid/limit

Study staff will be in contact with the subject weekly to facilitate dietary compliance once the dietary restrictions have begun.

6.2 Stabilization Initiation Visit 2 (Day -14)

Eligible subjects will return to the clinic in the morning for the following.

1. Review of medical history and concomitant therapies and current health status
2. Receive paper diaries for recording study product use, changes in concomitant therapies, physical activities, and any side effects/changes in current conditions will be recorded by the subject in this diary.

3. Receive 3-Day-Food records, which will be used to record all food and beverages consumed on two weekdays and one weekend day every week.
4. Receive dietary counselling from a dietician for the dietary restrictions for the study. These restrictions include:
    a. Avoiding foods containing high amounts of tryptophan and niacin;
    b. Limiting exposure to food containing moderate amounts of tryptophan and niacin;
    c. Avoiding foods and beverages known to contain NR during specified periods of time prior to and during the study. A list of these foods and beverages are provided below:
        i. Vitamins and health supplements containing niacin (nicotinic acid) or nicotinamide for 14 days prior to enrolment and during the study
        ii. Nutritional yeast (14 days prior to enrolment, during the study)
        iii. Whey proteins (14 days prior to enrolment, during the study)
        iv. Energy drinks (14 days prior to enrolment, during the study)

See Appendix 2 for a detailed list of food to avoid/limit

Study staff will be in contact with the subject weekly to facilitate dietary compliance.

5. The next visit will be scheduled after 14 days.

6.3 Visit 3 – Baseline (Day 0)

Eligible participants will return to the clinic in the morning, after fasting for 12 hours (nothing to eat or drink except water) for baseline assessments. Participants must not have consumed caffeine or exercised on the day of the visit, prior to the visit.

1. Review of medical history and concomitant therapies and current health status
2. A physical exam will be conducted
3. Participants will be randomized into the study.
4. Participants will be weighed and BMI will be calculated
5. Resting blood pressure and heart rate will be measured
6. REE will be evaluated
7. Study diaries and 3-Day Food Records will be collected and reviewed. Subjects will receive dietary counselling from a dietician.
8. Collect pre-dose urine sample for the measurement of Niagen™ metabolites and random urine creatinine analysis
9. Collect pre-dose blood for measurement of Niagen™ metabolite
10. Collect pre-dose blood samples for lipid panel, amino acid panel, hsCRP, AST, ALT, GGT and BUN
11. Collect muscle biopsy from the *Vastus Lateralis* of subject's leg for Niagen™ metabolite analyses.
12. New study diaries and 3-Day Food records will be dispensed to the participant. 3-Day Food records will be completed every week until the next visit, and the study diary will be completed daily.
13. Investigational product will be dispensed according to the randomization schedule.
14. After all assessments are completed the participant will take the first dose (4 capsules) of the product at the clinic.
15. Participants will be instructed in detail by site personnel about the treatment regimen.
16. The next visit will be scheduled for Day 7 this visit (± 3 days).

Study staff will be in contact with the subject weekly to facilitate dietary compliance.

6.4 Visit 4 and 5 (Days 7 and 14 ± 3 days)

Subjects will return to the clinic after fasting for 12 hours (nothing to eat or drink except water). Participants must not have consumed caffeine or exercised on the day of the visit, prior to the visit.

1. Review of medical history and concomitant therapies, current health status and adverse events
2. Unused investigational product in the original packaging and the remnants of the used packages will be collected and compliance will be calculated by counting the returned unused product.
3. Study diaries and 3-Day Food Records will be collected and reviewed.
4. New study diaries and 3- day food records will be dispensed. 3-Day Food records will be completed every week until the next visit, and study diaries will be completed daily until the next visit. Subjects will receive dietary counselling from a dietician.

5. Participant will be weighed and BMI will be calculated
6. Resting blood pressure and heart rate will be measured
7. REE will be evaluated
8. Collect blood for Niagen™ metabolite analysis
9. Collect urine for Niagen™ metabolite and random urine creatinine analyses
10. Collect blood samples for lipid panel, amino acid panel, hsCRP, AST, ALT, GGT and BUN

11. The next visit will be scheduled at the same time of day as previous visits (± 3 days).

Study staff will be in contact with the subject weekly to facilitate dietary compliance.

6.5 Visit 6 (Day 28 ± 3 days)

Subjects will return to the clinic after fasting for 12 hours (nothing to eat or drink except water). Participants must not have consumed caffeine or exercised on the day of the visit, prior to the visit.

1. Review of medical history and concomitant therapies, current health status and adverse events
2. Unused investigational product in the original packaging and the remnants of the used packages will be collected and compliance will be calculated by counting the returned unused product.
3. Study diaries and 3-Day Food Records will be collected and reviewed.
4. New study diaries and 3- day food records will be dispensed. 3-Day Food records will be completed every week until the next visit, and study diaries will be completed daily until the next visit. Subjects will receive dietary counselling from a dietician.

5. Participant will be weighed and BMI will be calculated
6. Resting blood pressure and heart rate will be measured
7. REE will be evaluated
8. Collect blood for Niagen™ metabolite analysis

9. Collect urine for Niagen™ metabolite and random urine creatinine analyses.
10. Collect blood samples for lipid panel, amino acid panel, CBC, electrolytes (Na, K, Cl), HbA1c, creatinine, bilirubin, hsCRP, AST, ALT, GGT and BUN

11. The next visit will be scheduled at the same time of day as previous visits (± 3 days).

Study staff will be in contact with the subject weekly to facilitate dietary compliance.

6.6 Visit 7- End of Study (Day 56 ± 3 days)

Subjects will return to the clinic after fasting for 12 hours (nothing to eat or drink except water). Participants must not have consumed caffeine or exercised on the day of the visit, prior to the visit.

1. Review of medical history and concomitant therapies, current health status and adverse events
2. Unused investigational product in the original packaging and the remnants of the used packages will be collected and compliance will be calculated by counting the returned unused product.
3. Study diaries and 3-Day Food records will be collected and reviewed
4. Subjects will be weighed and BMI will be calculated
5. Resting blood pressure and heart rate will be calculated
6. REE will be evaluated
7. Collect blood for Niagen™ metabolite analysis
8. Collect Urine for Niagen™ metabolite and random urine creatinine analyses
9. A muscle biopsy will be taken from the *Vastus Lateralis* of subjects leg for NAD metabolite analyses
10. Collect blood samples for lipid panel, amino acid panel, hsCRP, CBC, electrolytes (Na, K, Cl), HbA1c, creatinine, AST, ALT, GGT, BUN and bilirubin

6.7 Clinical Assessments and Procedures

Calculations or measurements of specific parameters are required as indicated in the schedule of assessments. Instructions for determining these parameters are provided in the following sections.

6.7.1 Height, Weight

Weight measurements should be performed with shoes removed, and bladder empty. Subjects should be weighed on the same scale at all visits.

At least two separate measurements should be taken at each visit. If the two measurements are more than 0.5 kg (1.1 lbs) apart, a third measurement should be taken. Then the two closest values are going to be selected and entered in the database.

Measurement of height should be performed with the subject's shoes removed. The subject's knees should be straightened, and head held upright.

6.7.2 Blood Pressure

In office, seated resting blood pressure and heart rate will be determined from 3 measurements obtained at least 1 minute apart. One arm will be chosen and used consistently throughout the study. Blood pressure will be checked in both arms at the first examination. If a consistent inter-arm difference exists, the arm with the higher pressure will be used throughout the study. The arm selected for use at the initial visit will be documented in the study file.

The subject should be seated comfortably with the back supported and the upper arm bared without restrictive clothing. Feet should be flat on the floor, legs should not be crossed. The subject should rest in this position for at least 5 minutes prior to the first reading.

The same recording method and the same equipment should be used for each subject throughout the study.

6.7.3 Micro-Needle Muscle Biopsy

Micro-needle muscle biopsies will be performed once at the Baseline visit (Visit 3, Day 0) once at the End of Study Visit (Visit 7, Day 56). Prior to the procedure, the Qualified Investigator, or designate, will ensure that the subject has completed the Muscle Biopsy Screening Form and has read and signed the Muscle Biopsy Information Sheet and fully understands the nature of the procedure and any associated risks.

The biopsy samples will be taken from the *Vastus Lateralis* muscle by an experienced physician or trained delegate. The surface area where the micro-needle will be inserted will be disinfected and anesthetized using a local anesthetic. A cannula will be used to pierce the skin and a micro-needle will be inserted. Following collection of the muscle sample, the needle will be removed and pressure will be applied to the area where the needle was inserted for a few minutes until any bleeding stops. The wound will then be treated and bandaged appropriately.

6.7.4 Indirect Calorimeter Analysis

Resting energy expenditure (REE, or resting metabolic rate; RMR) will be calculated at baseline and all subsequent visits to the clinic, until study completion. Subjects will come into the clinic having fasted for 12 hours and having avoided exercising and consuming caffeine on the day of testing, prior to the test. Subjects will be asked to place the ReeVue MetaBreather hose in their mouth and breathe normally. Participants will have nose clips placed on their nose for the duration of the analysis, which completes after ~10min. Subject REE data will be printed from the ReeVue machine and stored for further analysis.

6.7.5 Compliance

Investigational product compliance calculations will be necessary in this study because the products will be consumed at home. Subjects will be asked to bring any unused packages, the remains of the used packages, and their diaries to each study visit.

Compliance will be assessed by counting the returned unused study product at each visit. Compliance is calculated by determining the number of dosage units taken divided by the number of dosage units expected to have been taken multiplied by 100.

$$\frac{number\ of\ dosage\ units\ taken}{number\ of\ dosage\ units\ expected\ to\ have\ been\ taken} \times 100\%$$

In the event of a discrepancy between the information in the subject diary and the amount of study product returned, use will be based on the product returned unless an explanation for loss of

product has been provided. Subjects found to have a compliance of <80% or >120%will be counseled. A compliance of <70% or >130% will be considered as non-compliant and any subject demonstrating non-compliance for two consecutive visits will be withdrawn from the study.

Compliance to the protocol will also be assessed by reviewing the diaries completed by each subject for adherence to the study's dietary restrictions, ingestion of the investigational product, and maintenance of physical activity levels, and the determination of the urine Niagen^{TM} metabolites.

6.8 Laboratory Analyses

Blood samples will be drawn, urine will be collected, and muscle biopsies will be harvested from the subjects as indicated in the Schedule of Assessments. Subjects will be in a fasting state (last food intake at least 12 hours before the visit) prior to all of the blood draws except screening Additional blood samples may be collected during the course of the study in order to perform or repeat laboratory tests outlined in the Schedule of Assessments if needed.

Protection of subject confidentiality will extend to all data generated from the assaying of these samples. These samples will be alphanumerically coded and the persons performing the analysis will not be aware of the subject's identity.

At Visit 1 (screening), whole blood will be collected and dispensed into the following tubes:

1. Two 4 ml EDTA Tubes:
    a. One 4 ml EDTA tube will be used for CBC analysis;
    b. One 4 ml EDTA tube will be used to generate serum for HbA1C levels analysis;
2. One 5 ml SST tube for electrolyte (Na, K, Cl), creatinine, AST, ALT, GGT, BUN and bilirubin analyses.

At Visit 3 (baseline), whole blood, urine and a muscle biopsy will be collected and dispensed into the following tubes:

A. The whole blood will be dispensed into the following tubes:
   1. Two 2.7 ml light blue top citrate tubes for NAD analysis
   2. One 4ml EDTA Tube for NR metabolite analysis
   3. Two 5 ml SST tubes:
      a. One 5 ml SST tube will be used for lipid panel analysis and AST, ALT, GGT and BUN Analysis
      b. One 5 ml SST tube will be used for hsCRP analysis
   4. One 6 ml Green Top tube for Amino Acid analysis

B. The urine sample will be divided in 5 ml tubes for Niagen metabolite and creatinine analyses and snap frozen in liquid Nitrogen.

At Visit 4 and 5 (Day 7 and 14) whole blood and urine will be collected.

A. The whole blood will be dispensed into the following tubes:
   1. Two 5 ml SST tubes:
      a. One 5 ml SST tube will be used for hsCRP analysis;

b. One 5 ml SST tube will be used for AST, ALT, GGT and BUN analysis; and lipid panel analysis
2. One 6 ml Green Top tube for Amino Acid analysis
3. Two 2.7 ml light blue top citrate tubes for NAD analysis
4. One 4ml EDTA Tube for NR metabolite analysis

B. The urine sample will be divided in 5 ml tubes for Niagen metabolite and creatinine analyses and snap frozen in liquid Nitrogen.
C. The muscle biopsy collected at Visit 3 for Niagen metabolite analyses will be snap frozen in liquid nitrogen.

At Visit 6 (Day 28), whole blood and urine will be collected.

A. The whole blood will be dispensed into the following tubes:
1. Two 5 ml SST tubes:
   a. One 5 ml SST tube will be used for hsCRP analysis;
   b. One 5 ml SST tube will be used for electrolyte (Na, K, Cl), creatinine, AST, ALT, GGT and BUN analysis; and lipid panel analysis
2. Three 4 ml EDTA Tubes:
   a. One 4 ml EDTA tube will be used for NR metabolite analysis
   b. Two 4 ml EDTA tubes will be used for CBC and Hb1Ac analysis
3. One 6 ml green top tube will be used for amino acid panel analysis
4. Two 2.7 ml light blue top citrate tubes for NAD analysis

B. The urine sample will be divided in 5 ml tubes for Niagen metabolite and creatinine analyses and snap frozen in liquid Nitrogen.

At Visit 7 (Day 56), whole blood, urine and a muscle biopsy will be collected.

A. The whole blood will be dispensed into the following tubes:
1. Two 5 ml SST tubes
   a. One 5ml SST tube will be used for electrolytes (Na, K, Cl), creatinine, AST, ALT, GGT, BUN and bilirubin analysis; and lipid panel analysis
   b. One 5 ml SST tube will be used for hsCRP analysis.
2. One 6 ml green top tube will be used to generate plasma for amino acid panel analysis.
3. Three 4 ml EDTA tubes.
   a. One 4 ml EDTA tube will be used for CBC analysis
   b. One 4 ml EDTA tube will be used to generate serum for HbA1C analysis;
   c. One 4 ml EDTA tubes will be used for NR metabolite analysis

4. Two 2.7 ml light blue top citrate tubes for NAD analysis B. The urine sample will be equally divided into two 5 ml tube for Niagen metabolite and creatinine analyses and snap frozen in liquid Nitrogen.
C. The muscle biopsy collected for Niagen metabolite analyses will be snap frozen in liquid nitrogen.

For the analysis of NAD, the blood drawn in light blue top tubes will be immediately placed on ice. The tubes should be inverted 4 times then centrifuged at 2000 rpm at 4°C for 5 min. The plasma should be removed and aliquoted into two cryovials. The RBC fraction will be divided into 100uL portions by pipetting the cells into 4 chilled cryovial tubes containing 1ml of cold 0.5M perchloric acid. The tubes should be inverted 4 times then immediately frozen and stored at -80°C.

For the NR metabolite analysis, the blood drawn in EDTA tubes will be inverted and aliquoted into two 2ml cryovials and stored at -80°C.

Blood, muscle biopsies, and urine will be stored at -80°C and analyzed for Niagen™ metabolites (nicotinamide adenine dinucleotide (NAD), nicotinamide (Nam), N-methyl-2-pyridone-3/5-carboximide (2-PY), and methylnicotinamide (meNam)) at a laboratory of the sponsor's choosing. Urine samples will also be analyzed for creatinine; analysis of Niagen™ metabolites in urine will be standardized to creatinine concentrations.

Individuals performing the bioanalysis of study samples will remain blinded towards the dose of the investigational product that the participants receive.

The total blood volume collected for the laboratory assessments listed above will be approximately 156 ml over the period from screening to end of study. At any study visit, blood loss per subject is not expected to exceed approximately 33.4 ml.

LifeLabs Medical Laboratory Services in London, Ontario, Canada will be used in this study to measure safety laboratory parameters.

6.9 Termination of the Trial

In the case of premature termination of the trial, participating investigators/participants, and the Institutional Review Board must be promptly informed of the termination.

6.10 Protocol Amendments

If amendments to the study protocol are required after approval such changes will be captured in writing the reasons for the change documented and signed and dated by the sponsor. Any such amendments may be subject to IRB and Health Canada review/approval prior to implementation. Exception: if it becomes necessary to alter the protocol to eliminate an immediate hazard to patients, an amendment may be implemented prior to IRB approval. In this circumstance, the Investigator must notify IRB and Health Canada in writing within five (5) working days of the implementation.

7 Safety Instructions and Guidance

7.1 Adverse Events and Laboratory Abnormalities

7.1.1 Adverse Events

An adverse event (AE) is any untoward medical occurrence in a clinical investigation subject who has been administered an investigational product and which does not necessarily have a causal relationship with this treatment. An AE can be any unfavorable and unintended sign (including an abnormal laboratory finding), symptom, or disease temporally associated with the use of a product, whether or not it is considered related to that product. Pre-existing conditions which worsen during a study are to be reported as AEs.

During the study, subjects should record any adverse effects in their diary. At each visit the subject will be asked "Have you experienced any difficulties or problems since I saw you last"? Any adverse events (AEs) will be documented and in the study record and will be classified

according to the description, duration, intensity, frequency, and outcome. The investigator will assess any AEs and decide causality.

Intensity of AEs will be graded on a three-point scale (mild, moderate, severe) and reported in detail in the study record.

Mild:	Awareness of event but easily tolerated
Moderate:	Discomfort enough to cause some interference with usual activity
Severe:	Inability to carry out usual activity

The causality relationship of investigational product to the adverse event will be assessed by the investigator as either:

Most probable:	There is a reasonable relationship between the investigational product and AEs. The event responds to withdrawal of investigational product (dechallenge) and recurs with rechallenge when clinically feasible.
Probable:	There is a reasonable relationship between the investigational product and AEs. The event responds to dechallenge.
Possible:	There is a reasonable relationship between the investigational product and AEs. Dechallenge information is lacking or unclear.
Unlikely:	There is a temporal relationship to the investigational product administration but there is no reasonable causal relationship between the investigational product and the AEs.
Not related:	No temporal relationship to the investigational product administration or there is a reasonable causal relationship between non-investigational product, concurrent disease or circumstance and the AEs.

7.1.2 Serious Adverse Event

A serious adverse event (SAE) is any experience that suggests a significant hazard, contraindication, side effect or precaution. It is any AE that results in any of the following outcomes:

- Death
- A life-threatening adverse event
- Inpatient hospitalization or prolongation of existing hospitalization
- A persistent or significant disability or incapacity
- A congenital anomaly/birth defect in the offspring of a subject who received the study treatment
- Important medical events that may not be immediately life-threatening or result in death or hospitalization but may jeopardize the subject or may require intervention to prevent one of the outcomes listed above. Examples of such events are intensive treatment in an emergency room or at home for allergic bronchospasm; blood dyscrasias or convulsions that do not result in hospitalization; or the development of drug dependency or drug abuse.

7.1.3 Unexpected Adverse Reaction

An unexpected adverse reaction is an adverse reaction, the nature and severity of which is not consistent with the applicable product information (e.g., Investigator's Brochure for an unapproved

investigational product or package insert/summary of product characteristics for an approved product).

7.1.4 Laboratory Test Abnormalities

The investigator must assess the clinical significance of all abnormal laboratory values as defined by the compendium of normal values for the reference laboratory. Specific laboratory parameters include, hemoglobin concentration, hematocrit, WBC count, RBC count, platelet count, neutrophil count, lymphocyte count, monocyte count, eosinophil count, basophil count, fasting glucose levels, creatinine concentration, glomerular filtration rate, Na+/K+ concentration, Cl- concentration, total bilirubin, aspartate transaminase, alanine transaminase, and gamma-glutamyltransferase.

Any treatment emergent abnormal laboratory result which is clinically significant, i.e., meeting one or more of the following conditions, should be recorded as a single diagnosis on the AEs form in the study record:

- Accompanied by clinical symptoms
- Leading to interruption or discontinuation of the investigational product
- Requiring a change in concomitant therapy

This applies to any protocol and non-protocol specified laboratory result from tests performed after the first dose of the investigational product, which falls outside the laboratory reference range and meets the clinical significance criteria for liver and kidney tests as well as for hematology and clinical chemistry, etc. (i.e. AST and/or ALT > 2 x ULN).

This does not apply to any abnormal laboratory result which falls outside the laboratory reference range but which does not meet the clinical significance criteria or those which are a result of an AE which has already been reported.

Any laboratory result abnormality fulfilling the criteria for a serious adverse event (SAE) should be reported as such, in addition to being reported as an AE in the study record.

7.2 Treatment and Follow-up of AEs and Laboratory Abnormalities

7.2.1 Treatment and Follow-up of AEs

AEs, especially those for which the relationship to the investigational product is suspected, should be followed up until they have returned to baseline status or stabilized.

If after follow-up, return to baseline status or stabilization cannot be established, an explanation should be recorded in the study record.

7.2.2 Treatment and Follow-up of Laboratory Abnormalities

In the event of subject-initiated withdrawal or clinically significant unexplained abnormal laboratory test values, the subject will be withdrawn from the treatment and will remain in the study and be required to attend all remaining study visits as part of a safety arm.

7.3 Reporting of SAEs and Unexpected Adverse Reactions

The Qualified Investigator will be responsible for classification of an AE as an SAE within 24 hours of notification. Causality should be signed off by the Qualified Investigator prior to reporting to ethics and regulatory bodies. Notification of any serious adverse events must be made in writing to the study sponsor. The IRB will be notified of all SAEs and unexpected adverse reactions. All SAEs will be reported to the Therapeutics Products Directorate (TPD) in an expedited manner.

The sponsor must notify the TPD of all serious adverse reactions as follows:

a. If it is neither fatal or life threatening, within 15 calendar days after the day on which the sponsor becomes aware of the information; and

b. If it is fatal or life threatening, must be reported as soon as possible, but not later than seven (7) days after the day on which the sponsor becomes aware of the information.

7.4 Dietary monitoring

Throughout the study, the subject's diet will be monitored via 3-day food diaries dispensed at each study visit; subjects will receive dietary counseling from a dietician and a weekly phone call from study staff. The 3-day food diary should list all foods ingested during 2 weekdays and 1 weekend day. In addition, the ingestion of all tryptophan- and niacin (nicotinic acid and nicotinamide)-rich foods should be listed in the 3-day food record.

8 STATISTICAL EVALUATION

8.1 Determination of sample size

The planned sample size for this study is 140 subjects, with 35 subjects randomized to each treatment group. The primary efficacy endpoint is the change urinary methylnicotinamide levels from baseline to Day 56.

Power calculations were performed to determine the required sample size to provide 80% power at the 0.05 alpha level (that is, to have an 80% chance of obtaining $p \leq 0.05$ significance).

The sample sizes assume that (1) the basal concentration SD is equal to the end-of-study SD for the placebo group, and (2) the correlation coefficient between baseline and end-of-study values is equal to 0.5.

Based on a previous study by KGK Synergize, the standard deviation of the urinary methylnicotinamide levels was estimated to be 3.1μM/mM Creatinine. With an estimated 20% attrition over the course of this study, 80% power and $p \leq 0.05$ when comparing product to placebo, if the product produces at least a 2.5μM/mM Creatinine increase in urinary methylnicotinamide concentration, then a total of 128 subjects are required to be enrolled.

Based on a previous study by Fu et al. (1989), the standard deviation of the erythrocyte NAD levels was estimated to be 10.1μM. With an estimated 20% attrition over the course of this study, 80% power and $p \leq 0.05$ when comparing product to placebo, if the product produces at least an 8.7μM increase in erythrocyte NAD levels, then a total of 140 subjects are required to be enrolled.

Because erythrocyte NAD levels are a secondary endpoint in this study and require a larger sample size, the study was powered on the erythrocyte NAD concentration.

8.2 Analysis Plan

- The **Safety Population** will consist of all subjects who received any amount of either product, and on whom any post-randomization safety information is available.

- The **Intent-to-Treat (ITT) Population** consists of all subjects who received either product, and on whom any post-randomization efficacy information is available.
- The **Per Protocol (PP) Population** "Per Protocol (PP) Population" consists of all subjects who consumed at least 80% of treatment or placebo doses do not have any major protocol violations and complete all study visits and procedures connected with measurement of the primary variable.

8.3 Analysis Plan

An effectiveness analysis based on the modified intent-to-treat population and an efficacy analysis based on the per protocol population will be performed. Variables will be tested for normality and log-normality. Log-normally distributed variables will be analyzed in the logarithmic domain. Non-normal variables will be analyzed by appropriate non-parametric tests.

All missing values in the intent-to-treat (effectiveness) analysis will be imputed with the most recent previously-available value (LOCF, or "last-observation-carried-forward" imputation). No imputation will be performed for missing values of safety variables.

For each numerical endpoint, a summary table will prepared with a variety of summary statistics including mean, standard deviation, median, minimum value, and maximum value for each time point-treatment pairing. In order to account for any a priori differences between groups, the summary statistics of the changes from baseline will also be provided. For parameters requiring the logarithmic transformation, the summary statistics will be provided as non-transformed values. Mean values will be displayed as graphs, with a separate line for each product, and error bars indicating ± 1 SEM. Mean changes from baseline will be graphed similarly.

Statistical tests:
Numerical efficacy endpoints will be formally tested for significance between groups by Analysis of Covariance (ANCOVA). The dependent variable will be the value at each visit, the factor will be the treatment group, and the value at baseline (Day 0) will be the covariate. When the effect of treatment is significant (p-value ≤ 0.05), the pairwise Tukey-Kramer post-hoc test will be applied. Significant efficacy of the product, relative to placebo, will be inferred if the coefficient of the treatment group in the ANCOVA model is significantly different from zero (p≤0.05). Numerical endpoints that are intractably non-normal will be assessed by the Mann-Whitney U test. A within group analysis on efficacy endpoints will be done using the Student's paired t-test or, in the case of intractable non-normality, the Wilcoxon sign rank test.

Probabilities ≤ 0.05 will be considered statistically significant. All statistical analysis will be completed using the R Statistical Software Package Version 3.2.1 (R Core Team, 2015) for Microsoft Windows.

8.3.1 Premature Discontinuation Description

For each premature discontinuation, the following parameters will be listed: subject number, dates of start and end of treatment, and the reason of premature discontinuation.

8.3.2 Safety

For adverse events, a descriptive analysis will be given. Adverse events will be presented in a frequency table by category and treatment. Furthermore, description, frequency, severity and causality will be reported for each adverse event.

8.4 Protocol Deviation Description

Protocol deviations will be listed in the final study report.

8.5 Protocol Amendments

Once the protocol has been approved by the IRB and Health Canada, any changes to the protocol must be documented in the form of an amendment. All amendments will be documented in the final study report.

9 DATA COLLECTION AND STORAGE

All data collection and record storage will be done in compliance with ICH GCP Guidelines and applicable local regulatory guidelines.

10 ETHICAL ASPECTS OF THE STUDY

This study will be conducted with the highest respect for the individual participants (i.e., subjects) according to the protocol, the ethical principles that have their origin in the Declaration of Helsinki, and the ICH Harmonised Tripartite Guideline for GCP.

10.1 IRB Approval

KGK Synergize Inc. will supply relevant documents for submission to an IRB for the protocol's review and approval. This protocol; a copy of the informed consent form; and, if applicable, subject recruitment materials and/or advertisements and other documents required by all applicable laws and regulations; will be submitted to the IRB for approval. The IRB's written approval of the protocol and subject informed consent must be obtained before commencement of the study. The IRB approval must refer to the study by exact protocol title, number, and version date; identify versions of other documents (eg, informed consent form) reviewed; and state the approval date.

KGK must adhere to all requirements stipulated by the IRB. This may include notification to the IRB regarding protocol amendments, updates to the informed consent form, recruitment materials intended for viewing by subjects, local safety reporting requirements and submission of the investigator's annual/final status report to the IRB.

10.2 Subject Information and Informed Consent

Written consent documents will embody the elements of informed consent as described in the declaration of Helsinki and the ICH Guidelines for GCP and will be in accordance with all applicable laws and regulations. The informed consent form describes the planned and permitted uses, transfers, and disclosures of the subject's personal and personal health information for purposes of conducting the study. The informed consent form further explains the nature of the study, its objectives, and potential risks and benefits, as well as the date informed consent is obtained. The informed consent form will detail the requirements of the participant and the fact that he or she is free to withdraw at any time without giving a reason and without prejudice to his or her further medical care.

10.3 Potential Risks and Procedures to Minimize Risk

All potential risks are disclosed to study participants prior to their participation. The potential risks associated with this study include venipuncture and the associated risks. Risks associated with venipuncture include pain, bruising, and infection at the site. Alcohol swabs and proper venipuncture procedure will be followed to minimize the risk of infection.

11 QUALITY ASSURANCE AND QUALITY CONTROL

11.1 Auditing

All material used in clinical studies are subjected to quality control. Quality assurance audits may be performed by the sponsor or any health authority during the course of the study or after its completion.

The Investigator agrees to comply with the sponsor and regulatory requirements in terms of auditing of the study. This includes access to the source documents for source data verification.

11.2 Monitoring

An initiation meeting will be conducted by the sponsor or an approved representative (CRO). At this meeting, the protocol and logistical aspects of the study will be reviewed with the Investigator and all study staff.

Source documents will be reviewed to ensure that all items have been completed and that the data provided are accurate and obtained in the manner specified in the protocol. The subject files will be reviewed to confirm that:

- Informed consent was obtained and documented;
- Enrolled subjects fulfilled all inclusion criteria and did not meet any exclusion criteria;
- AE/SAE reporting has been performed as applicable;
- Study visits have been conducted as per protocol and information has been recorded in the appropriate place in the source document;
- The study product is being stored correctly and an accurate record of its dispensation to the study subjects is being maintained (accountability).

Incorrect, inappropriate, or illegible entries in the subject files will be returned to the Investigator or designee for correction. No data disclosing the identity of subjects will leave the study center. The Investigator and any designees will maintain confidentiality of all subject records.

The Investigator will permit trial-related monitoring, audits, IRB/IEC review, and regulatory inspections and will allow direct access to source data and documents for these purposes.

11.3 Data Management

Data required for the analysis will be acquired from source documentation (including laboratory reports) and entered into a Microsoft Office Access database designed specifically for this study. All data points entered into the study database are source data verified.

Protocol 15NRHC: A randomized, double-blind, placebo controlled parallel study investigating the effects of Niagen™ (Nicotinamide Riboside) on Niagen™ metabolites in healthy adults

High safety standards for the transfer and storage of study data are guaranteed by the use of technologies such as password protection, firewalls and periodic backup to protect stored data. Writing access to the system will be limited to authorized personnel.

All data is archived for a period not less than 25 years from the date of completion of the study in accordance with Health Canada regulatory requirements.

REFERENCES

14NBHC, K. S. I. (2015). A Randomized, Double-blind, Cross-Over Study of the Pharmacokinetics of Three Dosages of Niagen™ in Healthy Subjects.

Ref Type: Unpublished Work

Benyo, Z., Gille, A., Kero, J., Csiky, M., Suchankova, M. C., Nusing, R. M. et al. (2005). GPR109A (PUMA-G/HM74A) mediates nicotinic acid-induced flushing. *J Clin.Invest, 115,* 3634-3640.

Bogan, K. L. & Brenner, C. (2008). Nicotinic acid, nicotinamide, and nicotinamide riboside: a molecular evaluation of NAD+ precursor vitamins in human nutrition. *Annu.Rev.Nutr, 28,* 115-130.

Canto, C., Houtkooper, R. H., Pirinen, E., Youn, D. Y., Oosterveer, M. H., Cen, Y. et al. (2012). The NAD(+) precursor nicotinamide riboside enhances oxidative metabolism and protects against high-fat diet-induced obesity. *Cell Metab, 15,* 838-847.

Carlson, L. A. & Rosenhamer, G. (1988). Reduction of mortality in the Stockholm Ischaemic Heart Disease Secondary Prevention Study by combined treatment with clofibrate and nicotinic acid. *Acta Med Scand., 223,* 405-418.

Chi, Y. & Sauve, A. A. (2013). Nicotinamide riboside, a trace nutrient in foods, is a vitamin B3 with effects on energy metabolism and neuroprotection. *Curr.Opin.Clin.Nutr Metab Care, 16,* 657-661.

Fu, C. S., Swendseid, M. E., Jacob, R. A., & McKee, R. W. (1989). Biochemical markers for assessment of niacin status in young men: levels of erythrocyte niacin coenzymes and plasma tryptophan. *J Nutr., 119,* 1949-1955.

Imai, S. & Guarente, L. (2014). NAD+ and sirtuins in aging and disease. *Trends Cell Biol., 24,* 464-471.

Institute of Medicine (1998). Niacin. In Dietary Reference Intakes for Thiamine, Riboflavin, Niacin, Vitamin B6, Folate, Vitamin B12, Pantothenic Acid, Biotin, and Choline, ed. (Washington, DC: National Academy Press), pp. 123-149

Jacob, R.A., Swendseid, M.E., McKee, R.W., Fu, C.S., and Clemens, R.A. (1989). Biochemical markers for assessment of niacin status in young men: urinary and blood levels of niacin metabolites. J Nutr *119*, 591–598.

Karpe, F. & Frayn, K. N. (2004). The nicotinic acid receptor--a new mechanism for an old drug. *Lancet, 363,* 1892-1894.

Khan, N. A., Auranen, M., Paetau, I., Pirinen, E., Euro, L., Forsstrom, S. et al. (2014). Effective treatment of mitochondrial myopathy by nicotinamide riboside, a vitamin B3. *EMBO Mol.Med, 6,* 721-731.

12 APPENDICES

12.1 Appendix 1 Schedule of Assessments

Procedures/assessments	Visit 1<br>Screening<br>Day -28	Visit 2<br>Stabilization Initiation<br>Day -14	Visit 3<br>Baseline<br>Day 0	Visit 4<br>Week 1<br>Day 7	Visit 5<br>Week 2<br>Day 14	Visit 6<br>Week 4<br>Day 28	Visit 7<br>Week 8<br>Day 56
Informed consent	X						
Review inclusion/exclusion criteria	X						
Review medical history and current health status	X	X	X	X	X	X	X
Receive diet restrictions		X					
Urine pregnancy test	X						
Review concomitant therapies	X	X	X	X	X	X	X
Randomization			X				
Height*, weight, heart rate, blood pressure<br>**Height will only be measured at Visit 1*	X		X	X	X	X	X
Physical examination			X				
Laboratory test:<br>CBC*, electrolytes* (Na, K, Cl), HbA1c*, creatinine*, BUN, AST, ALT, GGT, and bilirubin*<br>**Only measured at Visit 1, 6 and 7*	X		X	X	X	X	X
Blood samples for NAD			X	X	X	X	X
Urine samples for NAD and creatinine			X	X	X	X	X
Muscle biopsy for NAD			X				X
REE analysis			X	X	X	X	X
Lipid Panel			X	X	X	X	X

Amino Acid Panel<br>**Only analyzed at Visit 3, 6, and 7. Other blood will be stored for future analysis.*			X	X	X	X	X
High sensitivity C-reactive Protein			X	X	X	X	X
Resting Metabolic Rate			X	X	X	X	X
Dietary Counseling from a dietician	X	X	X	X	X	X	
3-Day Food Records dispensed		X	X	X	X	X	
3-Day Food Records returned			X	X	X	X	X
IP dispensed			X	X	X	X	
IP returned				X	X	X	X
Treatment diary dispensed		X	X	X	X	X	
Treatment diary returned			X	X	X	X	X
Run-In diary dispensed		X					
Run-In diary returned			X				
Compliance calculated			X	X	X	X	X
Adverse events assessed				X	X	X	X

12.2 Appendix 2 Foods to Avoid/Limit during the Study

According to IOM, the 50% for ingestion of niacin equivalents/day is 40 mg/day for males and 30 mg/day for females. Our goal is to standardize the ingestion of niacin equivalents to approximately 20 mg/day throughout the study. 60 mg tryptophan equals one niacin equivalent. Subjects will be instructed to ensure that they are consuming greater than 16mg/day with the goal of approximately 20 mg/day.

Suggested Foods to Avoid

1. Poultry
2. Fortified breads and bread products
3. Fortified ready-made cereals
4. Energy drinks
5. Soy products (excluding tofu)
6. Other game

Nutritional counseling by a nutritionist will be provided on how to stay within 20 mg/day of niacin from the following food sources:

1. Beef
2. Pork
3. Eggs
4. Milk and dairy products
5. Seeds and nuts
6. Fish
7. Lobster
8. Crab

Niacin Counting

20 Niacin Equivalents (NE) maximum per day

Food	Serving Size	NE
**Dairy:**		
Milk 1%, 2%	1 cup	2
Cheese	50 g	2.7
	1 cup (shredded)	6.5
**Beef:**		
Lean Ground Beef	90 g	8.5
Steak	75 g	9.3
Salami	75 g	4.1
	1 slice (26 g)	1.4
Bologna (Baloney)	1 slice (28 g)	1.3
**Pork:**		
Pork Chop	75 g	7.3
Bacon	1 slice (8 g)	1.3
Ham	75 g	6.6
	1 slice (35 g)	3
Weiner (frankfurter)	1 weiner (38 g)	2
**Seafood:**		
Lobster	75 g	4.4
	1 cup	8.9
Crab	75 g	4.4
	1 crab leg (134 g)	7.8
Shrimp	75 g	5.6
	1 large shrimp (6g)	0.4
Salmon	75 g	11.1
Tuna	75 g	7.7
	1 can (172 g)	17.6
Haddock	75 g	6.9
Perch	75 g	5.2
Fish Fillet (Fast Food, Breaded)	1 fillet (91 g)	4.5
**Others:**		
Eggs	1 medium egg (46 g)	1.6
	1 cup (257 g, scrambled, omelet)	6.8
Tofu (extra firm)	150 g	3.4
	1 cup	6
Almonds	25 almonds or ¼ cup	2.8
Peanuts	60 ml or ¼ cup	6.4
Cashews	60 ml or ¼ cup	1.9
Walnuts	60 ml or ¼ cup	1.8

12.3 Appendix 3 Reference Range for Serum Amino Acids

Table 1. Plasma Amino Acid Age-Specific Reference Ranges (µmol/L)

Amino Acid	<1 month	1-23 months	2-17 years	Adults (≥18 years)
Aspartic acid	2-20	2-14	1-8	1-4
Glutamic acid	51-277	32-185	9-109	10-97
Hydroxyproline	13-72	7-63	6-32	4-27
Serine	87-241	83-212	85-185	65-138
Asparagine	12-70	20-77	23-70	31-64
α-Aminoadipic acid	≤3	≤4	≤2	≤2
Glycine	133-409	103-386	138-349	122-322
Glutamine	240-1194	303-1459	405-923	428-747
Sarcosine	≤5	≤4	≤4	≤4
β-Alanine	≤8	≤8	≤5	≤5
Taurine	29-161	26-130	32-114	31-102
Histidine	40-143	42-125	54-113	60-109
Citrulline	3-35	4-50	9-52	16-51
Arginine	14-135	30-147	38-122	43-407
Threonine	56-392	40-428	59-195	67-198
Alanine	83-447	119-523	157-481	200-483
1-Methylhistidine	≤4	≤9	≤27	≤47
γ-Aminobutyric acid	<1	<1	≤2	≤3
3-Methylhistidine	≤10	≤8	1-6	2-9
β-Aminoisobutyric acid	≤9	≤8	≤6	<1
Proline	87-375	104-348	99-351	104-383
Ethanolamine	8-106	5-19	5-15	5-13
α-Aminobutyric acid	1-20	4-30	6-30	7-32
Tyrosine[a]	33-160	24-125	31-108	38-96
Valine[a,b]	57-250	84-354	130-307	132-313
Methionine	13-45	12-50	14-37	16-34
Cystathionine	<1	<1	<1	<1
Isoleucine[a,b]	12-92	10-109	33-97	34-98
Leucine[a,b]	23-172	43-181	65-179	73-182
Homocystine	<1	<1	<1	<1
Phenylalanine[a]	30-79	31-92	38-86	40-74
Tryptophan[a]	17-85	16-92	30-94	40-91
Ornithine	29-168	19-139	33-103	27-83
Lysine	66-226	70-258	98-231	119-233
Alloisoleucine[b]	<1	<1	<1	<1

The full panel (test code 767X) includes all amino acids listed except alloisoleucine.
[a] Included in the limited panel (test code 1776X).
[b] Included in the MSUD panel (test code 19779X).

Amino acid panel source: Quest Diagnostics
