# Supplementary material for: Safety and Metabolism of Long-term Administration of NIAGEN (Nicotinamide Riboside Chloride) in a Randomized, Double-Blind, Placebo-controlled Clinical Trial of Healthy Overweight Adults
Source: Sci Rep. 2019 Jul 5;9:9772. doi: 10.1038/s41598-019-46120-z (PMC6611812; doi:10.1038/s41598-019-46120-z)
Supplement: Supplementary file 2 — Supplementary information [file 41598_2019_46120_MOESM2_ESM.docx]

Supplemental Data

Methods

The effect size and 95% confidence interval for the primary and secondary endpoints were determined using the effect size calculator provided by the Centre for Evaluation and Monitoring at Durham University (available at <https://www.cem.org/effect-size-calculator>, accessed on February 22, 2019).

Results

Table 1. Urinary 1-Methyl-Nicotinamide Levels in the Placebo- and NIAGEN^®^-Treated Groups at Day 56

| **Parameter** | **Placebo**  **(n=29)** | **100 mg NIAGEN**^®^  **(n=32)** | **300 mg NIAGEN**^®^  **(n=34)** | **1000 mg NIAGEN**^®^  **(n=31)** |
| --- | --- | --- | --- | --- |
| **Mean ± SD at Day 56**  **(ng MeNAM/ng creatinine)** | 4.1 ± 1.9^a^ | 6.6 ± 4.9^ab^ | 10.6 ± 16.1^b^ | 17.8 ± 8.8^c^ |
| **Effect Size** | NA | 0.66 | 0.55 | 2.12 |
| **95% Confidence Interval** | NA | 0.14, 1.17 | 0.03, 1.04 | 1.46, 2.72 |
| MeNAM, 1-methyl-nicotinamide; NA, not applicable; n, number; SD, standard deviation; ng, nanogram.  Numerical endpoints with different superscript letters are significantly different (p≤0.05) as assessed by Tukey-Kramer Pairwise test.  Note: At baseline 2 participants in the 100 mg NIAGEN^®^ group, six participants in the placebo group, 3 participants in the 300 mg NIAGEN^®^ group, and 2 participants in 1000 mg NIAGEN^®^ group had urine 1-meNAM concentrations below the assay detection levels of 1 μg/mL. At week 8, 1 participant in the 100 mg NIAGEN^®^ group and five participants in the placebo group had urine 1-meNAM concentration below the assay detection levels of 1 μg/mL. For participants who did not have end-of-study samples, their baseline values were not analyzed. Eight participants – 2 participants in the 100 mg NIAGEN^®^ group, 1 participant in the placebo group, 1 participant in the 300 mg NIAGEN^®^ group and 3 participants in the 1000 mg NIAGEN^®^ group – did not collect end-of-study samples because they did not complete the study. One participant in the 1000 mg NIAGEN^®^ group did not have a urine sample collected as their end-of-study visit was +14 days out of window and the participant was out of product for 14 days. For one participant in the 100 mg NIAGEN^®^ group urine creatinine concentration was not analyzed at baseline, therefore standardization could not be done and the baseline data of this participant was not included in the analysis. | | | | |

**Table 2. Rate of Change in Urinary 1-Methyl-Nicotinamide Levels in the Placebo- and NIAGEN^®^-Treated Groups from Baseline to Day 56**

| **Parameter** | **Placebo**  **(n=25)** | **100 mg NIAGEN**^®^ **(n=30)** | **300 mg NIAGEN**^®^ **(n=31)** | **1000 mg NIAGEN**^®^ **(n=29)** |
| --- | --- | --- | --- | --- |
| **Mean Rate of Change ± SD  Baseline to Day 56**  **(ng MeNAM/ng creatinine/day)** | 0.015 ± 0.032^a^ | 0.055 ± 0.085^ab^ | 0.125 ± 0.300^b^ | 0.259 ± 0.171^c^ |
| **Effect Size** | NA | 0.6 | 0.49 | 1.92 |
| **95% Confidence Interval** | NA | 0.05, 1.14 | 0.05, 1.02 | 1.25, 2.53 |
| MeNAM, 1-methyl-nicotinamide; NA, not applicable; n, number; SD, standard deviation; ng, nanogram.  Numerical endpoints with different superscript letters are significantly different (p≤0.05) as assessed by Tukey-Kramer Pairwise test.  Note: Eight participants – two participants in the 100 mg NIAGEN^®^ group, one participant in the placebo group, one participant in the 300 mg NIAGEN^®^ group and three participants in the 1000 mg NIAGEN^®^ group – did not collect Week 8 samples because they did not complete the study. In the 100 mg NIAGEN^®^ group two participants’ urine meNAM concentration was below the assay detection level of 1 µg/mL and one participant urine creatinine concentration was not analyzed therefore standardization could not be done at baseline and the data was not utilized. In the placebo group nine participants’ urine meNAM concentration was below the assay detection level of 1 µg/mL. Three participants in the 300 mg NIAGEN^®^ group and two participants in the 1000 mg NIAGEN^®^ group urine meNAM concentration was below the assay detection level of 1 µg/mL. One participant in the 1000 mg NIAGEN^®^ group did not have a urine sample collected as their end-of-study visit was +14 days out of window and the participant was out of product for 14 days. | | | | |

Table 3. Whole Blood NAD+ Levels in the Placebo and NIAGEN^®^-Treated Groups at Day 56

| **Parameter** | **Placebo**  **(n=34)** | **100 mg NIAGEN**^®^  **(n=33)** | **300 mg NIAGEN**^®^  **(n=34)** | **1000 mg NIAGEN**^®^  **(n=31)** |
| --- | --- | --- | --- | --- |
| **Mean ± SD at Day 56**  **(μg/ml)** | 21.0 ± 5.5^a^ | 24.3 ± 5.0^ab^ | 32.3 ± 9.9^b^ | 49.2 ± 22.7^c^ |
| **Effect Size** | NA | 0.63 | 1.41 | 1.74 |
| **95% Confidence Interval** | NA | 0.13, 1.11 | 0.86, 1.93 | 1.15, 2.29 |
| NAD+, nicotinamide adenine dinucleotide; NA, not applicable; n, number; SD, standard deviation; μg, microgram; ml, milliliter.  Numerical endpoints with different superscript letters are significantly different (p≤0.05) as assessed by Tukey-Kramer Pairwise test.  Note: Participants who did not have end-of-study blood samples were not analyzed. Eight participants – 2 participants in the 100 mg NIAGEN^®^ group, 1 participant in the placebo group, 1 participant in the 300 mg NIAGEN^®^ group and 3 participants in 1000 mg NIAGEN^®^ group – did not have end-of study blood samples collected because they did not complete the study. One participant in in the 1000 mg NIAGEN^®^ group did not have a blood sample collected for NAD+ analysis as their end-of-study visit was +14 days out of window and the participant was out of product for 14 days. One participant in 1000 mg NIAGEN^®^ group baseline value for NAD+ was not available. | | | | |

Table 4. Rate of Increase in Whole Blood NAD+ Levels in the Placebo- and NIAGEN^®^-Treated Groups from Baseline to Day 56

| **Parameter** | **Placebo**  **(n=34)** | **100 mg NIAGEN**^®^  **(n=33)** | **300 mg NIAGEN**^®^  **(n=34)** | **1000 mg NIAGEN**^®^  **(n=30)** |
| --- | --- | --- | --- | --- |
| **Mean Rate of Change ± SD  Baseline to Day 56**  **(μg/ml/day)** | -0.06 ± 0.13^a^ | 0.03 ± 0.10^a^ | 0.18 ± 0.17^b^ | 0.50 ± 0.39^c^ |
| **Effect Size** | NA | 0.77 | 1.59 | 1.98 |
| **95% Confidence Interval** | NA | 0.27, 1.26 | 1.02, 2.11 | 1.36, 2.55 |
| NAD+, nicotinamide adenine dinucleotide; NA, not applicable; n, number; SD, standard deviation; μg, microgram; ml, milliliter.  Numerical endpoint with different superscript letters are significantly different (p≤0.05) as assessed by Tukey-Kramer pairwise test.  Note: Participants who did not have end-of-study blood samples were not analyzed. Eight participants – 2 participants in the 100 mg NIAGEN^®^ group, 1 participant in the placebo group, 1 participant in the 300 mg NIAGEN^®^ group and 3 participants in the 1000 mg NIAGEN^®^ group – did not have end-of study blood samples collected because they did not complete the study. One participant in in 1000 mg NIAGEN^®^ group did not have a urine sample collected as their end-of-study visit was +14 days out of window and the participant was out of product for 14 days. One participant in the 1000 mg NIAGEN^®^ group Baseline value for NAD+ was not available. | | | | |

Table 5. Plasma Nicotinamide Levels in the Placebo and NIAGEN^®^-Treated Groups at Day 56

| **Parameter** | **Placebo**  **(n=34)** | **100 mg NIAGEN**^®^  **(n=33)** | **300 mg NIAGEN**^®^  **(n=34)** | **1000 mg NIAGEN**^®^  **(n=31)** |
| --- | --- | --- | --- | --- |
| **Mean ± SD at Day 56**  **(ng/ml)** | 22.3 ± 11.5^a^ | 26.6 ± 13.1^a^ | 27.9 ± 9.6^a^ | 43.7 ± 22.7^b^ |
| **Effect Size** | NA | 0.35 | 0.53 | 1.21 |
| **95% Confidence Interval** | NA | -0.14, 0.83 | 0.04, 1.01 | 0.66, 1.72 |
| NA, not applicable; n, number; Min, minimum; Max, maximum; SD, standard deviation; ng, nanograms; mL, milliliter.  Numerical endpoint with different superscript letters are significantly (p≤0.05) different as assessed by Tukey-Kramer Pairwise test.  Note: At baseline, 2 participants in the 100 mg NIAGEN® group, 1 participant in the Placebo group, 1 participant in the 300 mg NIAGEN^®^ group, and four participants in the 1000 mg NIAGEN^®^ group had missing values. At End-of-Study, 2 participants in the 100 mg NIAGEN^®^ group, 1 participant in the Placebo group, 1 participant in the 300 mg NIAGEN^®^ group, and four participants in the 1000 mg NIAGEN^®^ group have missing values. | | | | |

Table 6. Rate of Increase in Plasma Nicotinamide Levels in the Placebo- and NIAGEN^®^-Treated Groups

| **Parameter** | **Placebo**  **(n=34)** | **100 mg NIAGEN**^®^  **(n=33)** | **300 mg NIAGEN**^®^  **(n=34)** | **1000 mg NIAGEN**^®^  **(n=31)** |
| --- | --- | --- | --- | --- |
| **Mean Rate of Change ± SD  Baseline to Day 56**  **(ng/mL/day)** | 0.05 ± 0.20^a^ | 0.12 ± 0.21^a^ | 0.04 ± 0.52^a^ | 0.35 ± 0.52^b^ |
| **Effect Size** | NA | 0.34 | -0.03 | 0.76 |
| **95% Confidence Interval** | NA | 0.14, 0.82 | -0.50, 0.45 | 0.26, 1.24 |
| NA, not applicable; n, number; SD, standard deviation; ng, nanograms; mL, milliliter.  Numerical endpoint with different superscript letters are significantly (p≤0.05) different as assessed by Tukey-Kramer Pairwise test.  Note: At baseline, 2 participants in the 100 mg NIAGEN^®^ group, 1 participant in the Placebo group, 1 participant in the 300 mg NIAGEN^®^ group, and four participants in the 1000 mg NIAGEN® group had missing values. At End-of-Study, 2 participants in the 100 mg NIAGEN^®^ group, 1 participant in the Placebo group, 1 participant in the 300 mg NIAGEN^®^ group, and four participants in the 1000 mg NIAGEN^®^ group had missing values. | | | | |

Table 7. Plasma 1-Methyl-Nicotinamide Levels in the Placebo- and NIAGEN^®^-Treated Groups at Day 56

| **Parameter** | **Placebo**  **(n=34)** | **100 mg NIAGEN**^®^  **(n=33)** | **300 mg NIAGEN**^®^  **(n=34)** | **1000 mg NIAGEN**^®^  **(n=31)** |
| --- | --- | --- | --- | --- |
| **Mean ± SD at Day 56**  **(ng/ml)** | 3.1 ± 2.3^a^ | 5.6 ± 3.8^a^ | 10.1 ± 5.0^b^ | 26.6 ± 12.6^c^ |
| **Effect Size** | NA | 0.80 | 1.80 | 2.65 |
| **Precision** | NA | 0.29, 1.29 | 1.22, 2.34 | 1.96, 3.29 |
| NA, not applicable; n, number; SD, standard deviation; ng, nanogram; ml, milliliter.  Numerical endpoint with different superscript letters are significantly (p≤0.05) different as assessed by Tukey-Kramer Pairwise test.  Note: Below the quantification limit (BLQ) values were set to 1/2 of the lower limit of quantitation (LLOQ). | | | | |

Table 8. Rate of Increase in Plasma 1-Methyl-Nicotinamide Levels in the Placebo- and NIAGEN^®^-Treated Groups

| **Parameter** | **Placebo**  **(n=34)** | **100 mg NIAGEN**^®^  **(n=33)** | **300 mg NIAGEN**^®^  **(n=34)** | **1000 mg NIAGEN**^®^  **(n=31)** |
| --- | --- | --- | --- | --- |
| **Mean Rate of Change ± SD  Baseline to Day 56**  **(ng/ml/day)** | 0.009 ± 0.043^a^ | 0.050 ± 0.072^a^ | 0.125 ± 0.080^b^ | 0.429 ± 0.23^c^ |
| **Effect Size** | NA | 0.83 | 0.83 | 1.20 |
| **Precision** | NA | 0.32, 1.32 | -0.11, 0.85 | 0.66, 1.71 |
| NA, not applicable; N/n, number; SD, standard deviation; ng, nanogram; ml, milliliter.  Numerical endpoint with different superscript letters are significantly (p≤0.05) different as assessed by Tukey-Kramer Pairwise test.  Note: Below the quantification limit (BLQ) values were set to 1/2 of the lower limit of quantitation (LLOQ). | | | | |

**Table 9. Urinary N-Methyl-2-Pyridone-3/5-Carboximide in the Placebo and NIAGEN^®^-treated Groups at Day 56**

| **Parameter** | **Placebo**  **(n=34)** | **100 mg NIAGEN**^®^  **(n=33)** | **300 mg NIAGEN**^®^  **(n=34)** | **1000 mg NIAGEN**^®^  **(n=31)** |
| --- | --- | --- | --- | --- |
| **Mean ± SD at Day 56**  **(ng 2-PY/ng creatinine)** | 15 ± 7^a^ | 30 ± 24^ab^ | 51 ± 55^b^ | 113 ± 50^c^ |
| **Effect Size** | NA | 0.85 | 0.92 | 2.81 |
| **Precision** | NA | 0.34, 1.34 | 0.41, 1.41 | 2.09, 3.46 |
| NA, not applicable; n, number; 2-PY, N-methyl-2-pyridone-3/5-carboximide; SD, standard deviation, ng, nanogram.  Numerical endpoint with different superscript letters are significantly different (p≤0.05) as assessed by Tukey-Kramer pairwise test.  Note: Concentrations of 2-PY are standardized to creatinine concentrations in urine.  Participants who did not have end-of-study samples baseline values were not analyzed. Eight participants – two participants in the 100 mg NIAGEN^®^ group, one participant in the placebo group, one participant in the 300 mg NIAGEN^®^ group and three participants in the 1000 mg NIAGEN^®^ group – did not have end-of-study samples collected because they did not complete the study. One participant in the 1000 mg NIAGEN^®^ group did not have a urine sample collected as end-of-study visit was +14 days out of window and the participant was out of product for 14 days. One participant (C-D 108) in the 100 mg NIAGEN^®^ group urine creatinine concentration was not analyzed at baseline, so standardization could not be done and therefore the baseline data of this participant was not included. | | | | |

**Table 10. Rate of Increase in Urinary N-Methyl-2-Pyridone-3/5-Carboximide in the Placebo and NIAGEN^®^-treated Groups**

| **Parameter** | **Placebo**  **(n=34)** | **100 mg NIAGEN**^®^  **(n=33)** | **300 mg NIAGEN**^®^  **(n=34)** | **1000 mg NIAGEN**^®^  **(n=31)** |
| --- | --- | --- | --- | --- |
| **Mean Rate of Change ± SD  Baseline to Day 56**  **(ng 2-PY/ng creatinine/day)** | 0.02 ± 0.12^a^ | 0.28 ± 0.40^ab^ | 0.60 ± 0.99^b^ | 1.74 ± 0.93^c^ |
| **Effect Size** | NA | 0.89 | 0.82 | 2.66 |
| **Precision** | NA | 0.38, 1.39 | 0.32, 1.31 | 1.96, 3.29 |
| NA, not applicable; n, number; 2-PY, N-methyl-2-pyridone-3/5-carboximide; SD, standard deviation, ng, nanogram.  Numerical endpoint with different superscript letters are significantly different (p≤0.05) as assessed by Tukey-Kramer Pairwise test.  Note: Concentrations of 2-PY are standardized to creatinine concentrations in urine. Eight participants – 2 participants in the 100 mg NIAGEN^®^, 1 participant in the placebo group, 1 participant in the 300 mg NIAGEN^®^ group and 3 participants in the 1000 mg NIAGEN® group – did not have end-of-study samples collected because they did not complete the study. One participant in the 1000 mg NIAGEN^®^ group did not have a urine sample collected as their end-of-study visit was +14 days out of window and the participant was out of product for 14 days. | | | | |

Supplemental Figure


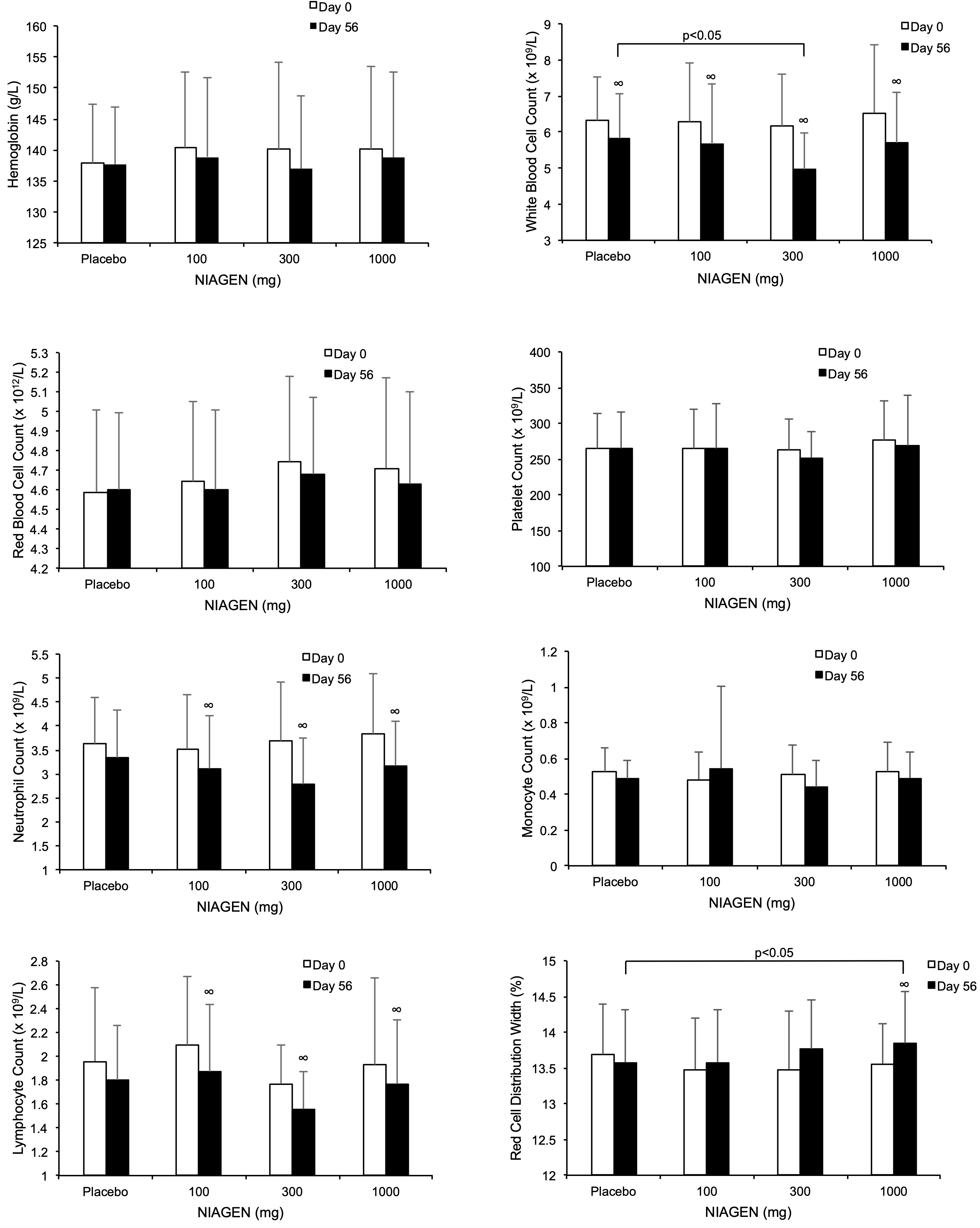


**NIAGEN supplementation does not adversely affect selected hematological parameters.** A, whole blood cell counts, B, mean corpuscular volume, C, mean corpuscular hemoglobin, D, red cell distribution widths, E, neutrophil counts, F, lymphocyte counts, G, monocyte counts, and H, basophil counts in the placebo- and NIAGEN-treated groups at Day 0 and 56. All data was obtained from Table 3. For whole blood cell counts, mean corpuscular volumes, mean corpuscular hemoglobin, red cell distribution widths, neutrophil counts, and lymphocyte counts, between-group comparisons at Day 0 and 56 were made using ANOVA and between-group comparisons adjusted for screening were made using ANCOVA. For the neutrophil and lymphocyte counts, logarithmic transformation was required to achieve normality. For the monocyte and basophil counts, between-group comparisons were made using the Kruskall-Wallis test and within-group comparisons were made using a non-parametric signed-rank test. Statistically significant (p<0.05) between group differences, which were determined using a Tukey-Kramer pairwise test, are highlighted with brackets. Statistically significant (p<0.05) within-group differences are highlighted with an infinity (∞) sign.
